# Co-Modified MnWO_4_ Nanorods Coupled with H_2_O_2_-Treated Carbon Nanotubes for a Charge-Balanced Aqueous Hybrid Supercapacitor

**DOI:** 10.3390/mi17080911

**Published:** 2026-07-29

**Authors:** Wei Xu, Changxu Qu, Jian Hao, Tingting Hao, Yabin Wang, Zheng Zhao, Yongnan Tan, Jing Wang

**Affiliations:** 1 School of Light Industry, Harbin University of Commerce, Harbin 150028, China; 2 State Key Laboratory of High-Efficiency Utilization of Coal and Green Chemical Engineering, Ningxia University, Yinchuan 750021, China; 3 School of Chemical Engineering, Harbin Institute of Technology, Harbin 150001, China; 4 College of Chemistry and Chemical Engineering, Yan’an University, Yan’an 716000, China; 5 Beijing Institute of Nonferrous Metals Research, Beijing 100088, China

**Keywords:** Co-modified MnWO_4_, H_2_O_2_-treated carbon nanotubes, aqueous hybrid supercapacitor, charge balance, battery-type electrode, combined-active-mass normalization

## Abstract

A charge-balanced aqueous hybrid supercapacitor was constructed by coupling Co-modified MnWO_4_ nanorods with H_2_O_2_-treated carbon nanotubes (OH-CNTs) in 1 M KOH. The samples were designated by the nominal Co/(Mn + Co) precursor molar fraction; ICP-OES measured bulk Co fractions of 0.09 ± 0.01, 0.47 ± 0.03, and 0.75 ± 0.04 mol% for the nominal 0.1, 0.5, and 0.8 mol% samples, respectively. Rietveld refinement confirmed retention of the monoclinic P2/c MnWO_4_ phase with only small composition-dependent lattice changes. At an active-material loading of 2.00 ± 0.05 mg cm^−2^, the optimized nominal 0.5 mol% sample delivered 429.5 ± 11.0 C g^−1^ (119.3 ± 3.1 mAh g^−1^) at 1 A g^−1^ and retained 280.5 ± 8.5 C g^−1^ (77.9 ± 2.4 mAh g^−1^) at 15 A g^−1^. Because the positive electrode exhibits battery-type behavior, specific charge and specific capacity are used as the primary performance descriptors. Over −0.9–0 V vs. SCE, pristine CNT and OH-CNT electrodes delivered of 189.0 ± 7.2 and 246.6 ± 8.1 C g^−1^, corresponding to electrode-level apparent specific capacitances of 215 ± 8 and 280 ± 9 F g^−1^ at 1 A g^−1^ after correction for the measured IR drop. These values apply to the stated 90:5:5 CNT (or OH-CNT)/acetylene-black/PVDF formulation and CNT-active-mass normalization and should not be interpreted as intrinsic capacitances of isolated commercial MWCNT powders. Charge matching based on the measured gave *m*^+^/*m*^−^ = 0.574; integration at the actual device loadings yielded *q*^+^ = 0.861 ± 0.015 C and *q*^−^ = 0.854 ± 0.015 C (*q*^+^/*q*^−^ = 1.008). The device operated over 0–1.6 V and delivered 97.9 ± 3.1 F g^−1^ at 0.51 A g^−1^, corresponding to 34.8 Wh kg^−1^ at 408 W kg^−1^ when normalized to the combined active mass of both electrodes. Three independently assembled devices retained 96.0 ± 0.7% of the initial capacitance and showed a coulombic efficiency of 98.8 ± 0.1% at the 10,000th cycle at 5.13 A g^−1^. Device metrics are normalized to the combined active mass of both electrodes and exclude current collectors, separator, electrolyte, and packaging.

## 1. Introduction

The increasing deployment of renewable-energy systems has intensified the need for safe electrochemical storage technologies capable of rapid power delivery and prolonged cycling [1]. Asymmetric supercapacitors extend the usable cell voltage by pairing electrodes with complementary potential windows, but their performance depends on the properties and mass balance of both electrodes [2]. Li et al. summarized how highly conductive two-dimensional materials can improve electron transport and interfacial accessibility [3]. Czagany et al. reviewed materials and electrode architectures for supercapacitors [4]; Sahin et al. and Navathe et al. discussed device design and practical operating considerations [5,6]; and Ma et al. focused on aging and degradation mechanisms [7]. More specifically, Li et al. showed that composition and oxygen-vacancy control can alter the electrochemical response of layered hydroxides [8], and Miao et al. demonstrated that conductive coupling and defect control are decisive in an aqueous hybrid device [9]. These studies indicate that credible device-level evaluation requires explicit reporting of electrode loading, potential window, reproducibility, charge balance, and normalization basis.

Transition-metal tungstates possess composition-sensitive metal–oxygen frameworks and accessible oxidation states and have therefore been investigated as Faradaic energy-storage materials [10,11]. For MnWO_4_–carbon systems, Sardar et al. reported 542.18 F g^−1^ at 2 mV s^−1^ for an MnWO_4_–amorphous-CNT hybrid [12], whereas Askari et al. reported 1849.14 F g^−1^ at 10 mV s^−1^ for an MnWO_4_–CNT electrode [13]. These CV-derived values were obtained under different loadings, current collectors, potential windows, and normalization conventions and are not directly comparable with GCD-derived specific charge at a stated current density. Recent studies further illustrate distinct structure–performance variables: hierarchical oxide morphology can shorten transport paths [14]; CoWO_4_ performance depends on morphology and synthesis conditions [15]; transition-metal modification of CoWO_4_/MXene changes interfacial transport [16]; Sn-doped WO_3_ exhibits temperature- and defect-dependent storage [17]; NiWO_4_ microflowers provide a high-accessibility architecture [18]; precursor concentration alters CoWO_4_ morphology and response [19]; composite tungstate design affects electrochemical utilization [20]; and a FeWO_4_/CNT device highlights the importance of pairing a tungstate positive electrode with a hydrophilic carbon negative electrode [21]. Collectively, these reports show that morphology, composition, pore accessibility, and device configuration must be evaluated together rather than inferred from a single gravimetric value.

Low-level precursor-composition control can modify nucleation, pore structure, redox-state distribution, and interfacial kinetics. Such effects have been observed in cobalt-modified hydroxides and oxides [22,23] and in cobalt-containing carbon-composite and core–shell electrodes [24,25,26]. However, precursor addition, measured bulk incorporation, crystallographic substitution, and electronic regulation are not equivalent claims, and direct evidence for a unique Co site in MnWO_4_ is not available here. The working hypothesis is therefore limited to a testable composition–structure–performance relationship: a moderate Co precursor fraction should produce reproducible changes in bulk composition, lattice parameters, morphology, surface redox states, pore accessibility, impedance, and charge storage kinetics while retaining the wolframite host phase. This hypothesis is evaluated against pristine MnWO_4_ using ICP-OES, Rietveld refinement, comparative XPS, porosimetry, and kinetic analysis.

The negative electrode is equally important because its stable potential range and accessible charge determine the positive/negative mass ratio and constrain the full-cell voltage. CNTs provide a conductive and mechanically robust network, but their hydrophobic surfaces can restrict wetting by aqueous electrolytes [27]. Electrochemical wetting and oxidative functionalization studies show that oxygen-containing groups can increase CNT surface polarity and electrolyte affinity [28,29]. Studies of carbon–metal-oxide and MnO_2_ systems provide qualitative context for oxygen-related surface reactions [30,31], but they do not establish the magnitude of a Faradaic contribution in a pure commercial CNT electrode. Accordingly, the nearly rectangular CV curves and triangular GCD profiles measured for the CNT-based negative electrodes indicate that charge storage is dominated by electric double-layer capacitance. The oxygen functionalities are therefore discussed primarily as improving aqueous-electrolyte wettability and utilization of the accessible carbon surface rather than as providing a significant pseudocapacitive contribution [32].

Accordingly, this study addresses three questions: whether controlled low-level Co modification produces reproducible structural and electrochemical differences relative to pristine MnWO_4_; whether H_2_O_2_ treatment increases CNT electrolyte accessibility while preserving the tubular conductive framework; and whether the independently evaluated electrodes can be quantitatively charge-matched to form a stable aqueous hybrid device. Positive-electrode performance is reported primarily as specific charge or specific capacity because the redox peaks and nonlinear GCD profiles indicate battery-type behavior; capacitance-equivalent values are used only as secondary descriptors. Full-cell capacitance, specific energy, and power are reported on the combined active mass of both electrodes, consistent with recommended electrochemical data-reporting practice [33]. The principal contribution is the integration of measured low-level Co composition, quantitative structural and surface-chemical comparison, pore and impedance analysis, kinetic evaluation across the complete MnWO_4_ series, CNT surface-chemistry modification, and experimentally verified electrode charge balance in one aqueous device. For the CNT negative electrode, the interpretation is limited to changes in measured surface oxygen, BET area, morphology, and electrode-level response under the stated preparation and testing conditions; no quantitative capacitance increment is assigned to a single oxygen-containing group.

## 2. Materials and Methods

Manganese chloride tetrahydrate (MnCl_2_·4H_2_O), cobalt chloride hexahydrate (CoCl_2_·6H_2_O), sodium tungstate dihydrate (Na_2_WO_4_·2H_2_O), ethylenediaminetetraacetic acid (EDTA), sodium hydroxide (NaOH), hydrogen peroxide (30 wt% H_2_O_2_), ethanol, and potassium hydroxide (KOH) were purchased from Sinopharm Chemical Reagent Co., Ltd. (Shanghai, China). Acetylene black, poly(vinylidene fluoride) (PVDF), N-methyl-2-pyrrolidone (NMP), nickel foam, and cellulose membranes were obtained from Guangdong Canrd New Energy Technology Co., Ltd. (Dongguan, China). Commercial multiwalled CNTs (>95% purity, 10–20 nm outer diameter, and 10–30 μm length) were purchased from Chengdu Organic Chemicals Co., Ltd., Chinese Academy of Sciences (Chengdu, China). All chemical reagents were of analytical grade and were used without further purification. Deionized water was used throughout the experiments.

### 2.1. Preparation of Co-MnWO_4_ Nanorods

**Figure 1:**
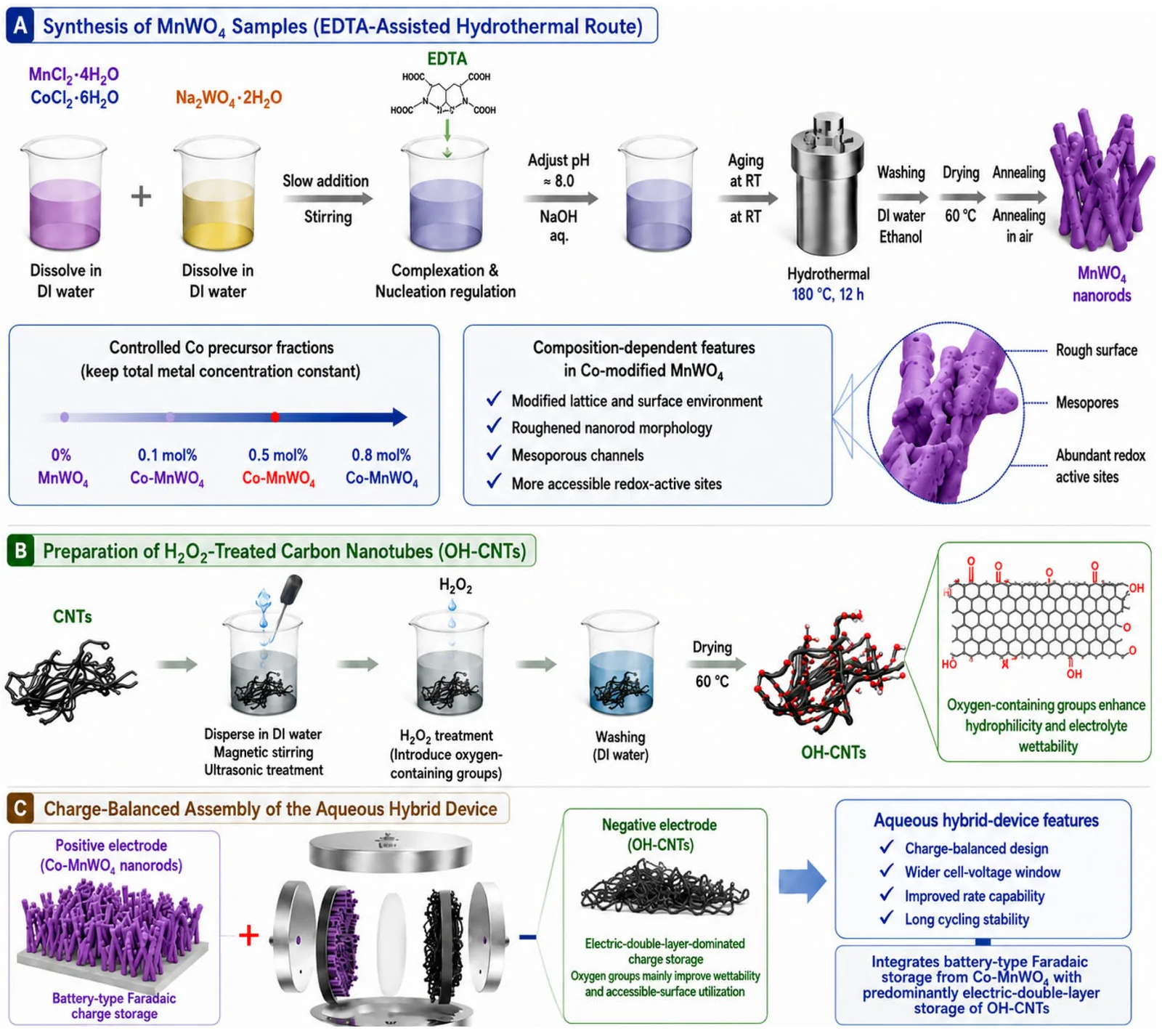
Schematic illustration of the synthesis and device design. (**A**) EDTA-assisted hydrothermal preparation of MnWO_4_ samples using controlled Co precursor fractions. (**B**) H_2_O_2_ treatment of CNTs to introduce oxygen-containing surface groups. (**C**) Charge-balanced assembly of the Co-MnWO_4_//OH-CNT aqueous hybrid device. The schematic represents battery-type Faradaic storage at the positive electrode and charge storage dominated by electric double-layer capacitance at the negative electrode; the oxygen functionalities mainly improve electrolyte wettability and utilization of the accessible carbon surface.

MnWO_4_ nanorods were synthesized using an EDTA-assisted hydrothermal route, as illustrated in Figure 1A. The nominal Co precursor fraction, x_nom_, was defined as x_nom_ = *n*(CoCl_2_·6H_2_O)/[*n*(MnCl_2_·4H_2_O) + *n*(CoCl_2_·6H_2_O)] × 100%, where n is the molar amount of the corresponding precursor. The total amount of Mn^2+^ and Co^2+^ was maintained at 5.000 mmol. For MnWO_4_ and the nominal 0.1, 0.5, and 0.8 mol% samples, the MnCl_2_·4H_2_O/CoCl_2_·6H_2_O masses were 989.53/0, 988.54/1.19, 984.58/5.95, and 981.61/9.52 mg, respectively. The metal precursors were dissolved in 30 mL of deionized water, and Na_2_WO_4_·2H_2_O (5.000 mmol, 1.649 g) was dissolved separately in 30 mL of deionized water. Both solutions were ultrasonicated for 8 min. The metal-ion solution was added to the tungstate solution over 20 min under continuous stirring. EDTA (5.000 mmol, 1.461 g) was introduced, and the pH was adjusted to 8.0 ± 0.1 using 1.0 mol L^−1^ NaOH. After aging for 50 min, the suspension was transferred to a 100 mL Teflon-lined autoclave (60% filling ratio), heated to 180 °C at 5 °C min^−1^, maintained for 12 h, and cooled naturally to room temperature over approximately 6 h. The precipitate was washed three times with deionized water and three times with ethanol, dried at 60 °C for 12 h, and annealed in air at 500 °C for 40 min using a heating rate of 5 °C min^−1^. Samples are denoted x mol% Co-MnWO_4_ according to x_nom_. ICP-OES measured bulk Co/(Mn + Co) fractions of <0.005, 0.09 ± 0.01, 0.47 ± 0.03, and 0.75 ± 0.04 mol% for MnWO_4_ and the nominal 0.1, 0.5, and 0.8 mol% samples, respectively.

### 2.2. Preparation of H_2_O_2_-Treated Carbon Nanotubes

OH-CNTs were prepared through controlled H_2_O_2_ oxidation, as shown in Figure 1B. CNTs (100 mg) were dispersed in 100 mL of deionized water by magnetic stirring for 30 min, followed by ultrasonication for 30 min at 300 W. H_2_O_2_ solution (50 mL, 30 wt%) was added dropwise over 30 min, and the suspension was treated at 60 °C for 6 h under continuous stirring with reflux. After cooling to room temperature, the product was collected by centrifugation at 8000 rpm for 10 min and washed with deionized water until the supernatant reached pH 6.8–7.2. The OH-CNTs were vacuum-dried at 60 °C for 12 h. The treatment was intended to improve surface polarity and electrolyte wettability while limiting disruption of the conductive tubular framework. In this manuscript, OH-CNT is retained solely as an operational sample label; the XPS results indicate a mixture of oxygen-containing groups, and the label neither implies an exclusively hydroxyl-terminated surface nor identifies a unique Faradaic charge-storage mechanism.

### 2.3. Electrode Preparation and Device Assembly

For the positive electrodes, MnWO_4_-based active material, acetylene black, and PVDF were mixed at a mass ratio of 80:10:10. Pristine CNT and OH-CNT negative electrodes were prepared using a CNT (or OH-CNT)/acetylene-black/PVDF ratio of 90:5:5. NMP was used as the slurry solvent; each mixture was ground for 30 min and magnetically stirred for 4 h. The slurries were deposited onto cleaned nickel foam with an exposed area of 1.0 cm^2^, vacuum-dried at 60 °C for 12 h, and pressed at 10 MPa for 30 s. The reported active-material loading and the mass used for gravimetric normalization refer only to MnWO_4_-based material or CNT material and exclude acetylene black and PVDF. The active-material loadings for MnWO_4_ and the nominal 0.1, 0.5, and 0.8 mol% samples were 1.96 ± 0.07, 1.98 ± 0.06, 2.00 ± 0.05, and 2.02 ± 0.06 mg cm^−2^, respectively; the pristine CNT and OH-CNT loadings were 1.88 ± 0.06 and 1.90 ± 0.05 mg cm^−2^. The response of blank nickel foam was measured under identical conditions and subtracted before gravimetric normalization. Three independently prepared electrodes were evaluated for each principal comparison. Acetylene black was not subtracted as a separate electrochemical blank; therefore, the stated formulation and normalization basis should be considered when comparing absolute gravimetric values. Accordingly, the CNT-based gravimetric values are electrode-formulation values and should not be interpreted as intrinsic capacitances of isolated commercial MWCNT powder. As a conservative cross-check, normalization to the total dry coating solids (active material, acetylene black, and PVDF) gives 343.6 C g^−1^ for the optimized positive electrode, 193.5 ± 7.2 and 252.0 ± 8.1 F g^−1^ for the pristine CNT and OH-CNT electrodes, respectively, and 84.3 F g^−1^ with 29.9 Wh kg^−1^ for the assembled device.

For device assembly, the nominal 0.5 mol% Co-MnWO_4_ sample was used as the positive electrode and OH-CNTs as the negative electrode. Charge balance was imposed using q^+^ = q^−^ and q = Q_s_m. With Q_s_^+^ = 429.5 C g^−1^ over 0–0.5 V vs. SCE and Q_s_^−^ = 246.6 C g^−1^ over −0.9–0 V vs. SCE, the calculated mass ratio was m^+^/m^−^ = 0.574. The positive and negative loadings in the 1.0 cm^2^ device were 2.00 and 3.48 mg cm^−2^, giving a combined active mass of 5.48 mg. At matched absolute current, integration of the discharge curves gave q^+^ = 0.861 ± 0.015 C and q^−^ = 0.854 ± 0.015 C (q^+^/q^−^ = 1.008), corresponding to a charge mismatch of 0.8%. A cellulose separator soaked in 1 M KOH was placed between the electrodes. Device current density, capacitance, specific energy, and power were normalized to m_total_ = m^+^ + m^−^.

### 2.4. Characterization

Morphology and microstructure were examined by field-emission SEM (15 kV), TEM/HRTEM (200 kV), and SAED. EDS spectra and elemental maps were acquired from three independently selected regions under identical acquisition conditions. Bulk Co content was quantified by ICP-OES after complete acid digestion. XRD patterns were collected with Cu Kα radiation over 10–80° 2θ using a step size of 0.02° and a scan rate of 2° min^−1^. Rietveld refinement was performed against the monoclinic P2/c MnWO_4_ structural model; lattice parameters, unit-cell volume, R_wp_, and χ^2^ were extracted from the refined profiles. XPS spectra of pristine MnWO_4_, nominal 0.5 mol% Co-MnWO_4_, pristine CNTs, and OH-CNTs were processed using a Shirley background and mixed Gaussian–Lorentzian line shapes. Binding energies were referenced to adventitious C 1s at 284.8 eV, and component ratios were calculated from integrated fitted areas using identical constraints for each comparison. Because the Co survey signal was low, the Co 2p area ratio was treated as semiquantitative and was not used as the sole basis for mechanistic conclusions. N_2_ adsorption–desorption measurements were performed at 77 K after degassing at 120 °C for 12 h; BET surface area, total pore volume, mesopore volume, and BJH average pore diameter were obtained from the adsorption data. Nanorod diameters were measured from 100 rods selected across multiple micrographs and independently imaged regions.

### 2.5. Electrochemical Measurements and Calculations

Single-electrode electrochemical measurements were conducted in 1 M KOH at 25 ± 2 °C using a three-electrode configuration. The active-material-coated electrode, a Pt sheet, and a saturated calomel electrode (SCE) served as the working, counter, and reference electrodes, respectively. The same reference electrode was used for all positive- and negative-electrode measurements. The operating windows were 0–0.5 V vs. SCE for MnWO_4_-based positive electrodes and −0.9–0 V vs. SCE for CNT-based negative electrodes. These limits were selected by stepwise CV/GCD screening, excluding ranges that produced an abrupt current upturn, an additional irreversible feature, or pronounced discharge-profile distortion. CV, GCD, and EIS measurements were performed using a CHI 760E workstation. EIS spectra were collected from 100 kHz to 0.01 Hz using a 5 mV perturbation and were fitted using the same R_s_–(R_ct_∥CPE)–W circuit and weighting procedure for all replicate electrodes, where W denotes a Warburg diffusion element; fit quality was evaluated using the fitted χ^2^ value. The assembled device was evaluated over 0–1.6 V after independent full-cell voltage-window screening. The separately selected three-electrode windows were used for electrode characterization and charge matching, whereas the full-cell window was established empirically in the two-electrode configuration, in which polarization is redistributed between the charge-balanced electrodes. Because individual electrode potentials were not monitored during full-cell operation, 1.6 V is reported only as a device-level operating window. Device currents of 2.80, 8.44, 14.04, 22.48, 28.13, and 42.17 mA corresponded to combined-active-mass current densities of 0.51, 1.54, 2.56, 4.10, 5.13, and 7.69 A g^−1^, respectively. Long-term device cycling was conducted at 5.13 A g^−1^ and 25 ± 2 °C. Unless otherwise stated, quantitative electrochemical values are mean ± standard deviation from three independently prepared electrodes or devices. EIS and kinetic parameters were fitted independently for each replicate.

Q_s_ = IΔt/m (1)

C_s_ = IΔt/(mΔV_eff_) = Q_s_/ΔV_eff_ (2)

q = Q_s_m = C_s_mΔV_eff_ (3)

m^+^/m^−^ = Q_s_^−^/Q_s_^+^ (4)

C_device_ = IΔt/(m_total_ΔV_cell,eff_) (5)

E = I∫V(t)dt/(3.6m_total_) (6)

P = 3600E/Δt(7)

where I is the discharge current, Δt is the discharge time, m is the active mass of the tested electrode, and ΔV_eff_ is the effective discharge interval after subtraction of the measured initial IR drop. Q_s_ is the gravimetric in C g^−1^ and is converted to mAh g^−1^ by division by 3.6. For the battery-type MnWO_4_-based electrodes, Q_s_ and mAh g^−1^ are the primary performance descriptors; C_s_ is reported only as an explicitly identified capacitance equivalent calculated from Q_s_/ΔV_eff_. For the CNT-based electrodes, C_s_ was calculated using the corresponding IR-drop-corrected discharge interval. Charge matching was performed directly from Q_s_, so it is independent of how capacitance equivalents are expressed. For full-cell analysis, m_total_ = m^+^ + m^−^ is the combined active mass of both electrodes. Energy was recalculated by numerical integration of the measured discharge curve using Equation (6), and power was calculated on the same mass basis. The near-linear GCD profile permits the conventional C_device_ΔV^2^/7.2 expression as a cross-check, but the integrated value is used for reporting. These active-material-level metrics exclude current collectors, separator, electrolyte, and packaging. The initial IR drops at 1 A g^−1^ were 26 ± 2, 24 ± 2, 19 ± 2, and 22 ± 2 mV for MnWO_4_ and the nominal 0.1, 0.5, and 0.8 mol% samples, respectively; the corresponding pristine CNT and OH-CNT values were 22 ± 2 and 18 ± 2 mV. For kinetic analysis, i = av^b^ was fitted over 5–100 mV s^−1^, and surface-controlled and diffusion-associated contributions were separated using i(V) = k_1_v + k_2_v^1/2^ following the Dunn approach [34]. For device cycling, coulombic efficiency was defined as *η* = *Q*_discharge_/*Q*_charge_ × 100%, where *Q*_charge_ and *Q*_discharge_ are the charge and discharge quantities of the same cycle.

## 3. Results and Discussion

### 3.1. Synthesis Strategy and Structural Design

Figure 1 summarizes the experimental design. Controlled nominal Co precursor fractions were introduced during EDTA-assisted hydrothermal growth of MnWO_4_, while H_2_O_2_ treatment was used to modify CNT surface chemistry and electrolyte affinity. EDTA was employed as a complexing agent and is expected, on the basis of prior coordination chemistry, to influence precursor complexation and nucleation. Because an EDTA-free control was not included, its specific mechanistic role was not experimentally isolated and is not presented as a demonstrated cause of the observed morphology. The two electrodes were evaluated separately and subsequently combined using quantitative charge balance. Detailed synthesis conditions are provided in Section 2.

### 3.2. Morphology and Elemental Distribution of Co-MnWO_4_

**Figure 2:**
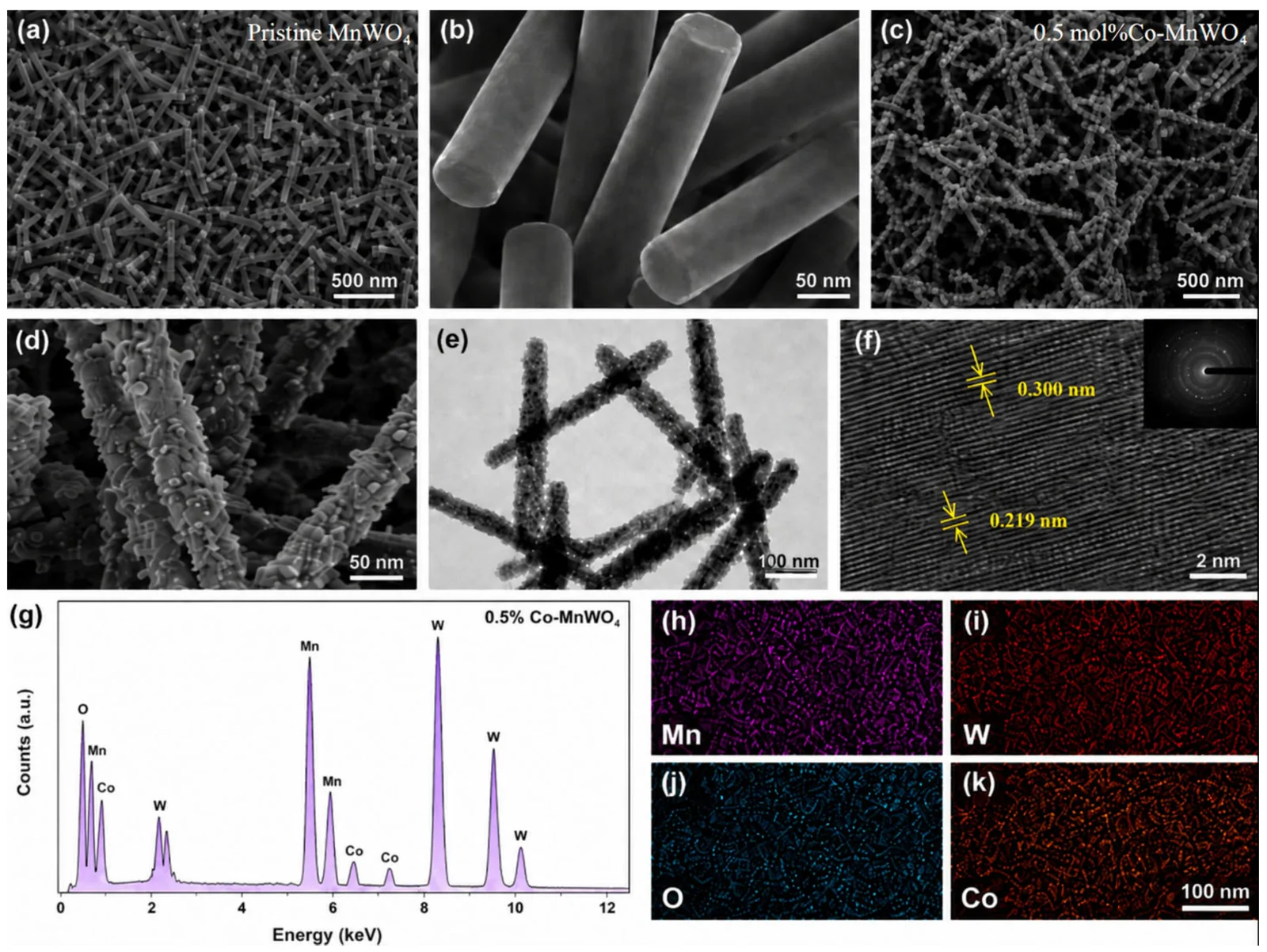
Morphology and elemental distribution of pristine MnWO_4_ and 0.5 mol% Co–MnWO_4_. (**a**,**b**) SEM images of pristine MnWO_4_. (**c**,**d**) SEM images of the Co-modified sample. (**e**) TEM image of the interconnected nanorod network. (**f**) HRTEM image and SAED inset; lattice fringes of approximately 0.300 and 0.219 nm are indexed to the (11−1) and (20−1) planes, respectively. (**g**) EDS spectrum and (**h**–**k**) elemental maps of Mn, W, O, and Co. Local EDS quantification from three independently selected regions gives Co/(Mn + Co) = 0.57 ± 0.06 at.%, while ICP-OES gives a bulk Co/(Mn + Co) fraction of 0.47 ± 0.03 mol%. The elemental maps indicate spatial overlap of Co with the MnWO_4_ nanorods without resolved Co-rich domains.

Figure 2 compares pristine MnWO_4_ with the sample prepared using a nominal Co/(Mn + Co) precursor molar fraction of 0.5 mol% (0.47 ± 0.03 mol% by ICP-OES). Both samples retain an interlaced nanorod morphology. Image analysis of 100 rods gives average diameters of 52 ± 9 nm for pristine MnWO_4_ and 58 ± 11 nm for the Co-modified sample. The modified nanorods display more visible nanoscale surface protuberances in the examined SEM fields; this is a qualitative morphological observation rather than an AFM-derived roughness measurement. It is therefore interpreted only together with the independently measured BET surface area and pore-volume data. The TEM image confirms an interconnected nanorod network without obvious bulk agglomeration.

The HRTEM image in Figure 2f shows dominant lattice fringes of approximately 0.300 and 0.219 nm. Based on the refined monoclinic P2/c cell, these spacings are indexed to the (11−1) and (20−1) planes, respectively. The EDS spectrum confirms Mn, W, O, and Co, and the elemental maps show spatial overlap of Co with the nanorods without resolved Co-rich domains. Semiquantitative analysis of three regions gives Co/(Mn + Co) = 0.57 ± 0.06 at.% and Co/Mn = 0.0057 ± 0.0006, in reasonable agreement with the bulk ICP-OES value of 0.47 ± 0.03 mol%. These measurements establish the presence and microscale homogeneous distribution of low-level Co; they do not by themselves identify a unique crystallographic substitution site.

### 3.3. Crystal Structure and Surface Chemical States of Co-MnWO_4_

**Figure 3:**
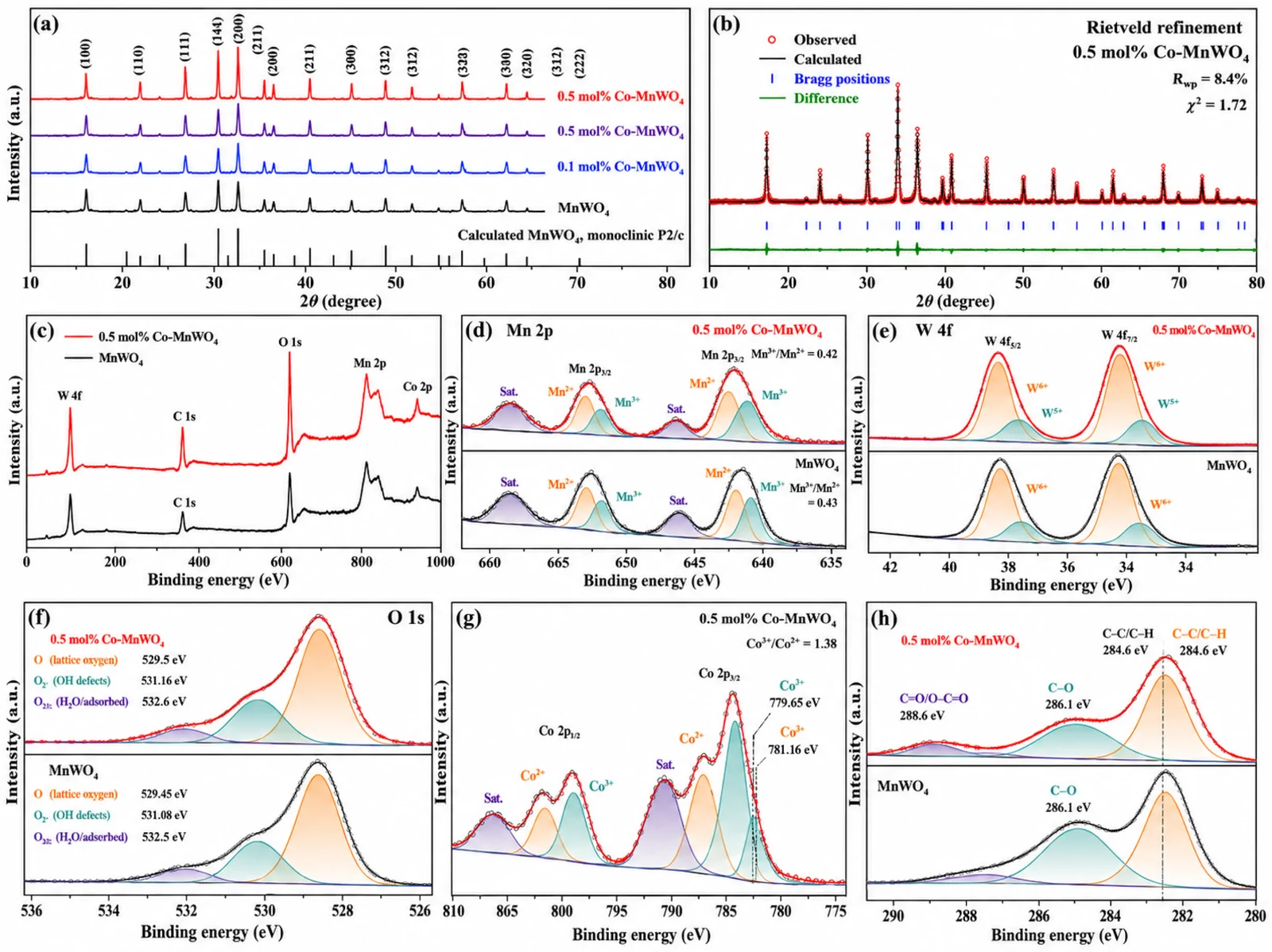
Crystal structure and surface chemical states of pristine and Co-modified MnWO_4_. (**a**) XRD patterns with indexed reflections. (**b**) Representative Rietveld refinement and difference curve for the nominal 0.5 mol% Co-MnWO_4_ sample; numerical refinement results for all four compositions are reported in the text. (**c**) Comparative XPS survey spectra. (**d**–**f**) Comparative Mn 2p, W 4f, and O 1s spectra. (**g**) Co 2p spectrum of 0.5 mol% Co-MnWO_4_, with Co^3+^ assigned at lower binding energy than Co^2+^. (**h**) C 1s spectra used for charge referencing at 284.8 eV. Quantitative parameters are summarized in Table 1 and Table 2.

The XRD patterns of MnWO_4_ and the Co-modified samples are shown in Figure 3a. All resolved reflections are indexed to monoclinic wolframite-type MnWO_4_ (space group P2/c), and no crystalline impurity phase is detected. The calculated peak positions and d-spacings from the refined structure are listed in Table 1. A representative refined profile for the nominal 0.5 mol% sample is shown in Figure 3b, while the numerical refinement results for all four compositions are reported below. Independent Rietveld refinements give a/b/c/β/V values of 4.8291(6) Å/5.7583(7) Å/4.9962(6) Å/91.146(12)°/138.904 Å^3^ for MnWO_4_; 4.8293(6)/5.7585(7)/4.9964(6) Å/91.148(12)°/138.920 Å^3^ for nominal 0.1 mol% Co-MnWO_4_; 4.8300(6)/5.7592(7)/4.9970(6) Å/91.153(12)°/138.973 Å^3^ for nominal 0.5 mol% Co-MnWO_4_; and 4.8304(7)/5.7595(8)/4.9973(7) Å/91.157(13)°/139.000 Å^3^ for nominal 0.8 mol% Co-MnWO_4_. The corresponding R_wp_ values are 8.4, 8.2, 7.9, and 8.3%, with χ^2^ values of 1.42, 1.38, 1.31, and 1.40. The changes are small and should not be interpreted as stand-alone proof of a particular Co site. Together with ICP-OES and the absence of a resolved impurity phase, the refinement supports composition-dependent modification of the MnWO_4_ host while leaving the detailed site occupancy unresolved.

XPS was used to compare pristine MnWO_4_ and nominal 0.5 mol% Co-MnWO_4_ using identical background and line-shape constraints; the comparative survey and high-resolution spectra are shown in Figure 3c–h. Survey quantification excluding adventitious carbon gives Mn/W/O atomic fractions of 16.1/16.4/67.5% for MnWO_4_ and 15.9/16.2/67.8%, with 0.10 at.% Co, for the modified sample. The Mn^2+^ and Mn^3+^ 2p_3/2_ components shift from 640.30 and 641.65 eV to 640.48 and 641.82 eV, respectively; the W^6+^ and W^5+^ 4f7/2 components shift from 34.90 and 33.78 eV to 35.02 and 33.90 eV. The Mn^3+^/Mn^2+^ ratio increases from 0.32 to 0.47, and W^5+^/W^6+^ increases from 0.08 to 0.14. The fitted O 1s fractions (lattice oxygen/OH- or defect-related oxygen/adsorbed oxygen or water) change from 65.2/23.6/11.2% to 57.4/29.8/12.8%. These assignments are consistent with prior MnWO4/tungstate and mixed-valence oxide analyses [10,11,12,13,35]. In the Co 2p_3/2_ envelope, Co^3+^ and Co^2+^ are located at 779.65 and 781.05 eV, respectively, with a satellite near 786.2 eV; the lower binding energy assigned to Co^3+^ corrects the previous deconvolution [35]. Because the Co survey concentration is only 0.10 at.%, the fitted Co^2+^/Co^3+^ area ratio of 1.38 is treated as semiquantitative. C 1s spectra used for charge referencing at 284.8 eV are included in Figure 3h. The comparative shifts and fitted area ratios are consistent with a modified surface redox environment; however, given the small binding-energy shifts and low Co signal, these XPS results do not by themselves establish a unique bulk-substitution or electronic-regulation mechanism. The quantitative XPS fitting parameters are summarized in Table 2.

### 3.4. Pore Structure and Electrolyte-Accessible Surface

**Figure 4:**
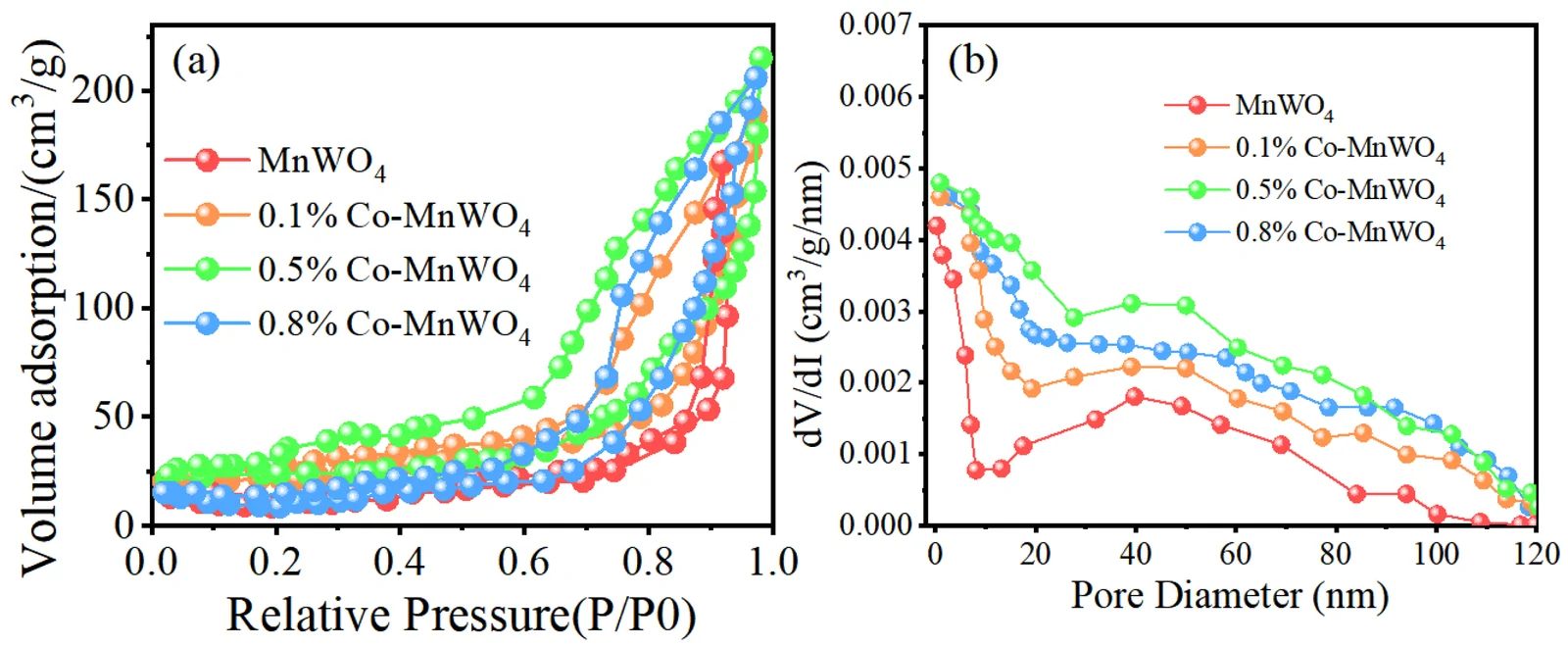
N_2_ adsorption–desorption characterization of MnWO_4_ and samples prepared with nominal Co/(Mn + Co) precursor fractions of 0.1, 0.5, and 0.8 mol%. (**a**) Type-IV adsorption–desorption isotherms with H3 hysteresis loops and (**b**) BJH pore-size distributions. BET surface area, total pore volume, mesopore volume, and average pore diameter are reported in Table 3.

The N_2_ adsorption–desorption curves in Figure 4a exhibit type-IV isotherms with H3-type hysteresis loops at high relative pressure, indicating mesoporous structures associated with slit-like voids and interparticle channels. The corresponding BJH distributions in Figure 4b confirm predominantly mesoporous transport pathways. The measured BET surface area, total pore volume, mesopore volume, and average BJH pore diameter are summarized in Table 3. The mesopore volumes of MnWO4 and the nominal 0.1, 0.5, and 0.8 mol% Co-modified samples are 0.21, 0.25, 0.30, and 0.27 cm^3^ g^−1^, respectively.

The 0.5 mol% Co-MnWO_4_ sample exhibits the largest BET surface area (72.4 m^2^ g^−1^), mesopore volume (0.30 cm^3^ g^−1^), specific charge at 1 A g^−1^ (429.5 ± 11.0 C g^−1^), and the lowest R_ct_ (3.2 ± 0.2 Ω). Across the series, the specific charges are 354.0 ± 9.0, 383.0 ± 10.0, 429.5 ± 11.0, and 406.0 ± 10.5 C g^−1^ (98.3 ± 2.5, 106.4 ± 2.8, 119.3 ± 3.1, and 112.8 ± 2.9 mAh g^−1^), broadly following the surface-area and mesopore-volume trend. This correlation supports improved electrolyte accessibility and utilization of redox-active sites, while the XPS and impedance results indicate that surface chemistry and charge-transfer kinetics also contribute.

### 3.5. Electrochemical Performance of Co-MnWO_4_ Positive Electrodes

**Figure 5:**
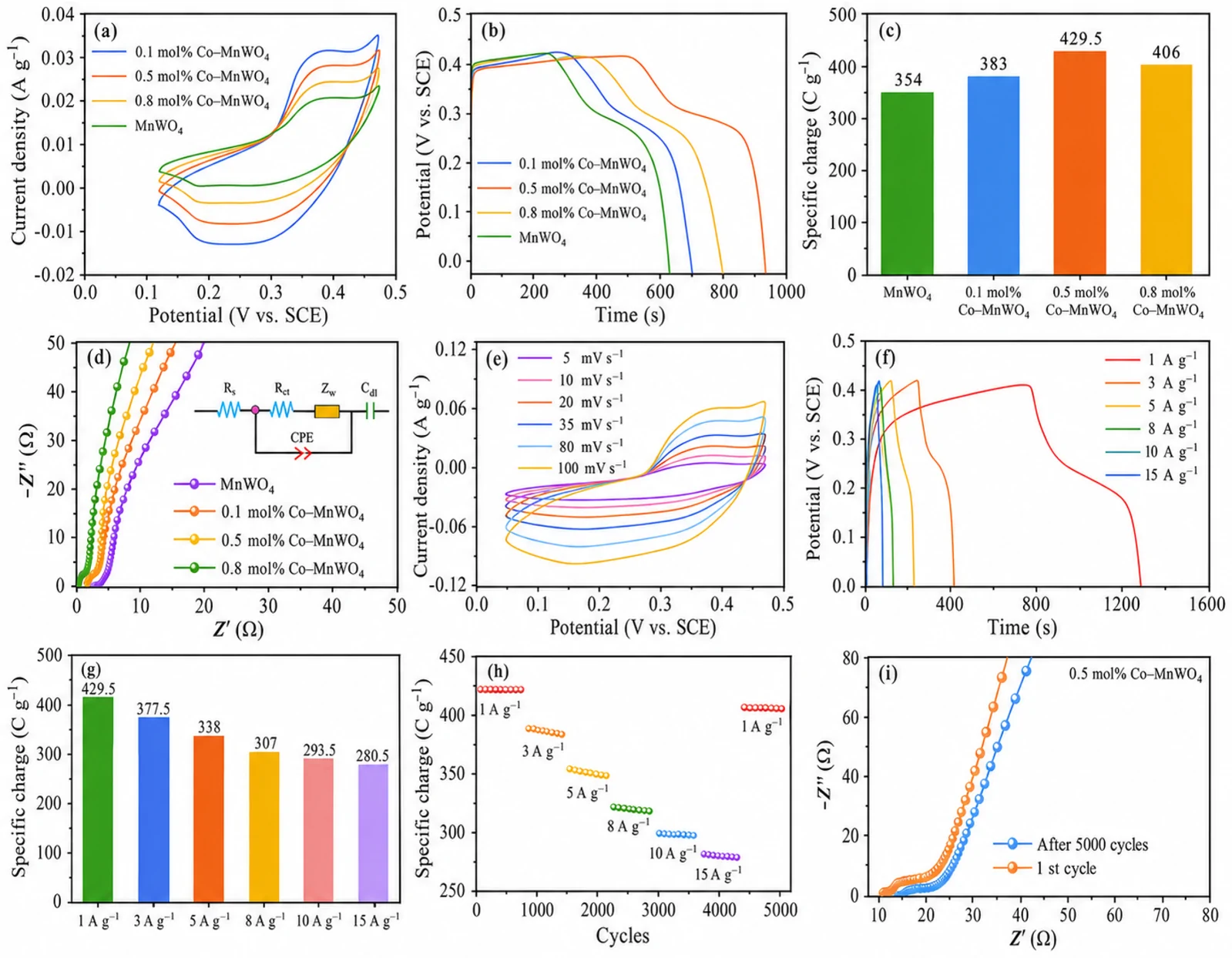
Electrochemical performance of MnWO_4_ and Co-modified positive electrodes in 1 M KOH. (**a**) CV curves showing Faradaic redox peaks. (**b**) GCD curves under identical conditions. (**c**) IR-drop-corrected capacitance-equivalent comparison at 1 A g^−1^: 747 ± 19, 805 ± 21, 893 ± 23, and 849 ± 22 F g^−1^ for MnWO_4_ and the nominal 0.1, 0.5, and 0.8 mol% samples, respectively; specific charge/capacity are the primary metrics. (**d**) Nyquist plots and equivalent circuit; fitted parameters are listed in Table 4. (**e**) CV curves of nominal 0.5 mol% Co-MnWO_4_ at 5–100 mV s^−1^. (**f**) GCD curves at 1–15 A g^−1^. (**g**) Specific-charge rate response. (**h**) Sequential rate-cycling response. (**i**) EIS before and after 5000 cycles at 10 A g^−1^. Gravimetric values are mean ± standard deviation (*n* = 3) and normalized to 2.00 ± 0.05 mg cm^−2^ active material.

The electrochemical behavior of MnWO_4_ and Co-modified positive electrodes was evaluated in a three-electrode configuration. Figure 5a shows pronounced oxidation and reduction peaks with a limited rectangular background, indicating predominantly Faradaic, battery-type charge storage rather than ideal electric-double-layer or purely pseudocapacitive behavior. The 0.5 mol% Co-MnWO4 sample exhibits the largest enclosed CV area and the longest GCD discharge time under identical test conditions (Figure 5a,b). This comparison identifies the best-performing sample within the investigated series, while the composition, structure, pore, impedance, and kinetic results are considered together when discussing the origin of the improvement.

Figure 5c summarizes the GCD-derived specific charges at 1 A g^−1^: 354.0 ± 9.0, 383.0 ± 10.0, 429.5 ± 11.0, and 406.0 ± 10.5 C g^−1^ for MnWO_4_ and the nominal 0.1, 0.5, and 0.8 mol% samples, respectively, equivalent to 98.3 ± 2.5, 106.4 ± 2.8, 119.3 ± 3.1, and 112.8 ± 2.9 mAh g^−1^. The active-material loadings are 1.96 ± 0.07, 1.98 ± 0.06, 2.00 ± 0.05, and 2.02 ± 0.06 mg cm^−2^, and the initial IR drops are 26 ± 2, 24 ± 2, 19 ± 2, and 22 ± 2 mV. Using the corresponding effective discharge intervals of 0.474, 0.476, 0.481, and 0.478 V gives IR-drop-corrected capacitance equivalents of 747 ± 19, 805 ± 21, 893 ± 23, and 849 ± 22 F g^−1^, respectively. These F g^−1^ values are mathematical equivalents over a narrow voltage interval and are not used to classify the electrodes as ideal capacitors; specific charge and capacity remain the primary metrics.

The Nyquist plots in Figure 5d were fitted using an equivalent circuit containing R_s_, R_ct_, a constant-phase element (CPE), and a Warburg diffusion element (W). Mean fitted parameters from three independently prepared electrodes are summarized in Table 4. The nominal 0.5 mol% Co-MnWO_4_ electrode gives the lowest R_ct_ (3.2 ± 0.2 Ω), compared with 5.9 ± 0.3, 4.7 ± 0.2, and 4.0 ± 0.2 Ω for MnWO_4_ and the nominal 0.1 and 0.8 mol% samples, respectively. Its lower R_s_ and Warburg coefficient are consistent with reduced internal resistance and more favorable ion transport. The fitted χ^2^ values of 1.3–2.1 × 10^−3^ indicate internally consistent fits under the common circuit model; the comparison is interpreted within this series rather than as an absolute mechanistic assignment.

The CV and GCD rate series in Figure 5e–g retain the main redox features of the nominal 0.5 mol% sample from 5 to 100 mV s^−1^, while the nonlinear GCD profiles confirm Faradaic battery-type storage. The specific charges are 429.5 ± 11.0, 377.5 ± 10.5, 338.0 ± 9.5, 307.0 ± 9.0, 293.5 ± 8.5, and 280.5 ± 8.5 C g^−1^ at 1, 3, 5, 8, 10, and 15 A g^−1^, respectively, equivalent to 119.3 ± 3.1, 104.9 ± 2.9, 93.9 ± 2.6, 85.3 ± 2.5, 81.5 ± 2.4, and 77.9 ± 2.4 mAh g^−1^. The specific-charge retention is 65.3% at 15 A g^−1^. Capacitance-equivalent values are not used for the rate series because the IR drop changes with current density. The rate-cycling sequence in Figure 5h demonstrates recovery when the current returns to 1 A g^−1^. The EIS comparison in Figure 5i, before and after 5000 cycles at 10 A g^−1^ over 0–0.5 V vs. SCE, indicates a moderate increase in interfacial impedance, supporting stable operation under the stated laboratory conditions.

### 3.6. Three-Electrode Testing Configuration

**Figure 6:**
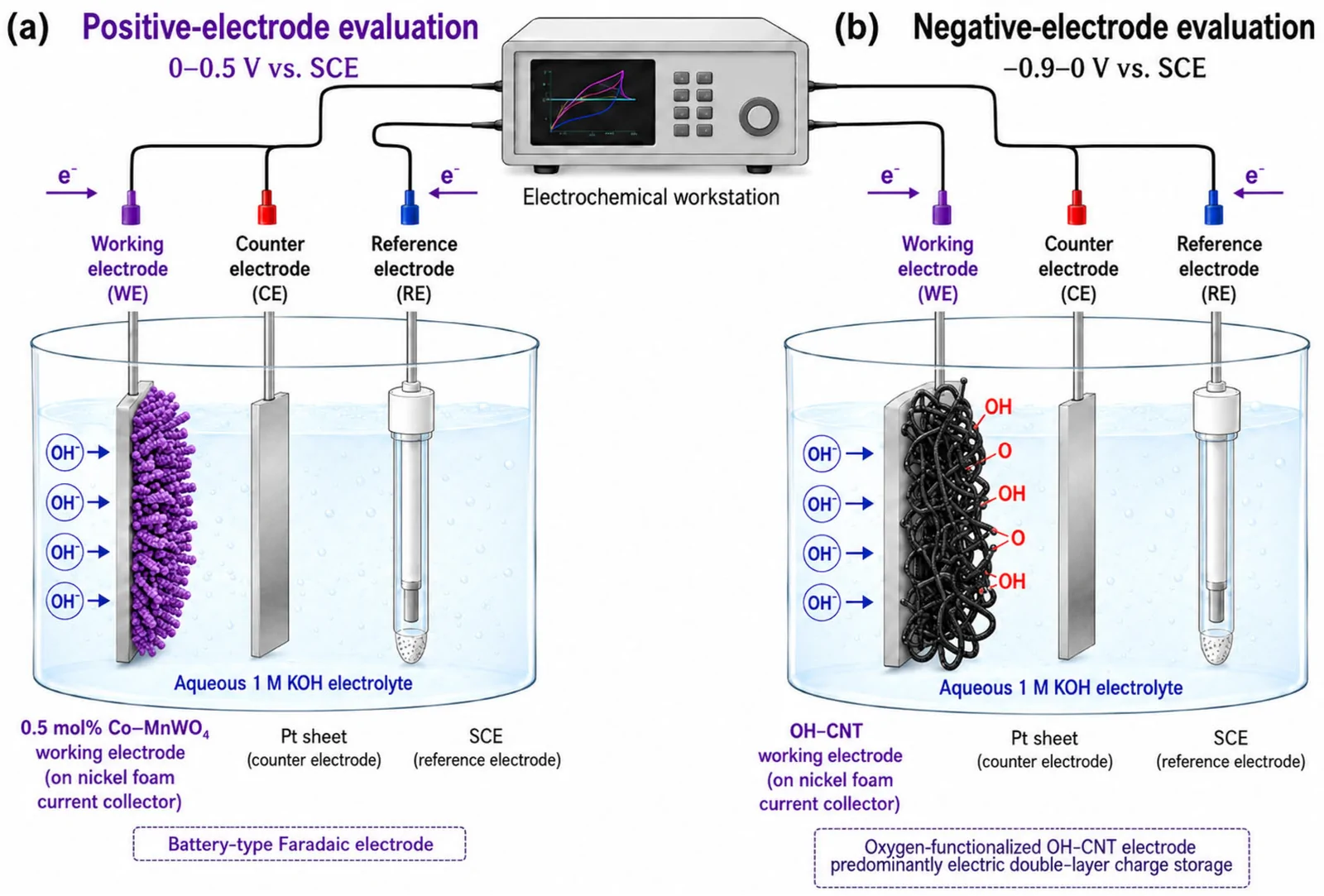
Three-electrode configurations used to evaluate the positive and negative electrodes in 1 M KOH. The active-material-coated electrode, Pt sheet, and SCE served as the working, counter, and reference electrodes, respectively. The same reference electrode was used for both electrode types.

The electrochemical testing configuration is illustrated in Figure 6. The 0.5 mol% Co-MnWO_4_ and OH-CNT electrodes were evaluated separately as working electrodes in 1 M KOH using a Pt-sheet counter electrode and an SCE reference electrode. The same reference system was used for both electrode types. Their independently measured stable potential windows, specific charges/capacitances, and loadings were used for charge matching and for defining the device voltage window.

### 3.7. Comparative Charge-Storage Kinetics of MnWO_4_ and Co-Modified Electrodes

Charge-storage kinetics were analyzed for pristine MnWO_4_ and all Co-modified electrodes using i = av^b^ over 5–100 mV s^−1^. The anodic/cathodic b-values are 0.68 ± 0.02/0.62 ± 0.02 for MnWO_4_, 0.74 ± 0.02/0.69 ± 0.02 for nominal 0.1 mol% Co-MnWO_4_, 0.82 ± 0.02/0.76 ± 0.02 for nominal 0.5 mol% Co-MnWO_4_, and 0.77 ± 0.02/0.71 ± 0.02 for nominal 0.8 mol% Co-MnWO_4_. The larger b-values of the optimized sample indicate a greater surface-controlled contribution than pristine MnWO_4_. In every sample, the cathodic b-value is lower than the anodic value, consistent with stronger diffusion and polarization constraints during reduction. Across the four anodic/cathodic b fits and the corresponding peak-current–v^1/2^ fits, the individual coefficients of determination fall within the sample-specific ranges listed in Table 5 (minimum R^2^ ≥ 0.992).

**Figure 7:**
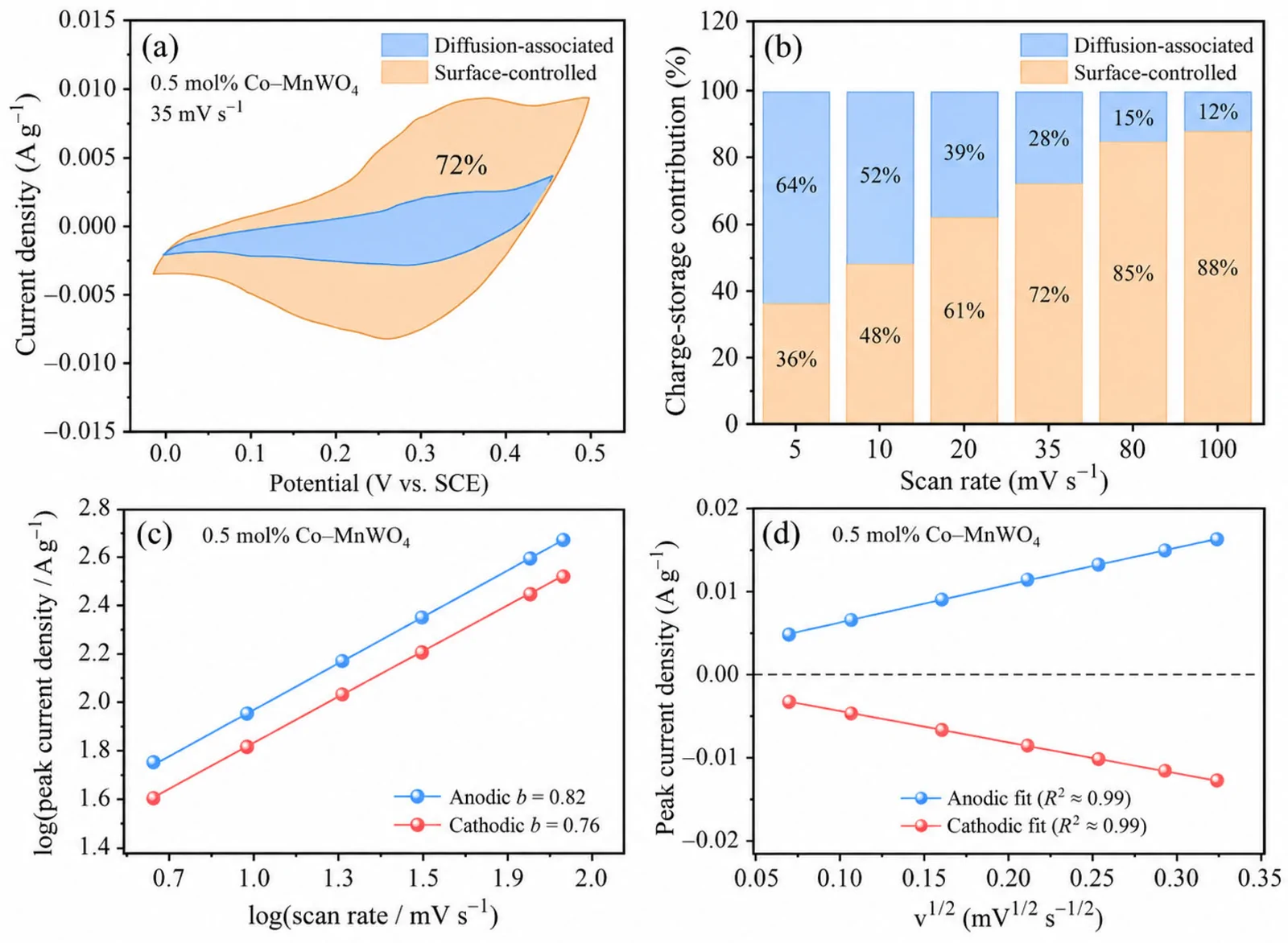
Kinetic analysis of nominal 0.5 mol% Co-MnWO_4_. (**a**) Separation at 35 mV s^−1^, showing 72 ± 2% surface-controlled and 28 ± 2% diffusion-associated contributions. (**b**) Contribution fractions at 5–100 mV s^−1^. (**c**) log(i)–log(v) fits with anodic and cathodic b-values of 0.82 ± 0.02 and 0.76 ± 0.02. (**d**) Peak-current versus v^1/2^ fits. Comparative values and fitting-quality ranges for all MnWO_4_-based electrodes are reported in Table 5.

The current response was further separated using i(V) = k_1_v + k_2_v^1/2^. At 35 mV s^−1^, the surface-controlled fractions of MnWO_4_ and the nominal 0.1, 0.5, and 0.8 mol% Co-MnWO_4_ samples are 54 ± 2, 63 ± 2, 72 ± 2, and 66 ± 2%, respectively; the complementary diffusion-associated fractions are 46 ± 2, 37 ± 2, 28 ± 2, and 34 ± 2%. Thus, the 72% surface-controlled and 28% diffusion-associated contributions in Figure 7a are the values at 35 mV s^−1^ and are consistent with the scan-rate series in Figure 7b. The optimized composition combines the largest surface-controlled fraction with a substantial diffusion-associated Faradaic contribution, explaining its balance of charge storage and rate response without implying a purely capacitive mechanism.

### 3.8. Structure and Surface Chemistry of OH-CNT Negative Electrodes

**Figure 8:**
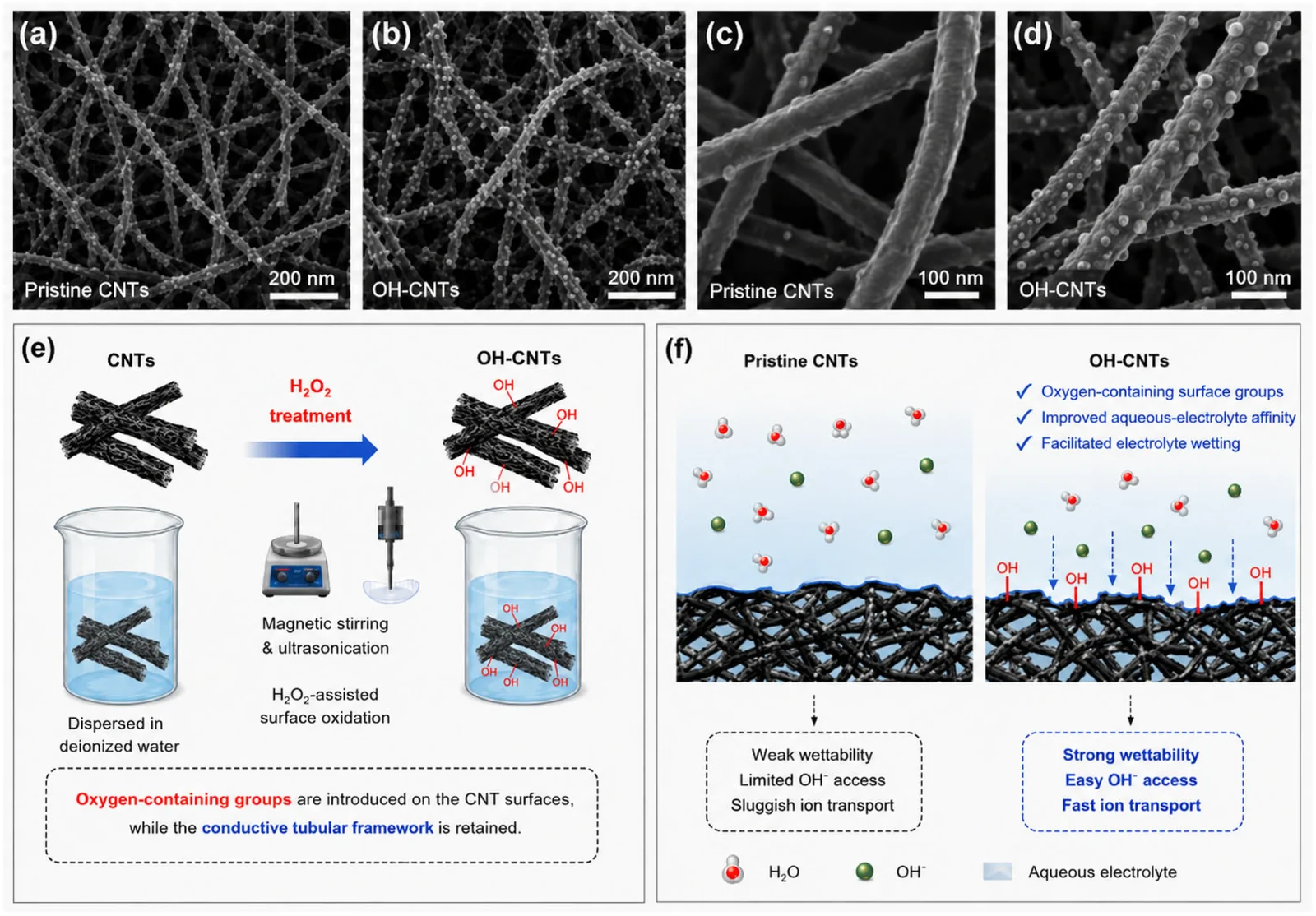
SEM images and schematic representation of H_2_O_2_ treatment of CNTs. (**a**,**b**) Low-magnification SEM images of pristine CNTs and OH-CNTs. (**c**,**d**) Higher-magnification SEM images showing preservation of the tubular network and a more irregular surface after treatment. (**e**) H_2_O_2_-treatment scheme. (**f**) Proposed influence of oxygen-containing groups on aqueous-electrolyte affinity. The schematic is conceptual; surface chemistry is quantified by XPS, and the SEM images are not used as direct evidence of specific functional groups.

Figure 8 compares pristine CNTs and OH-CNTs. Both samples retain an entangled tubular network after H_2_O_2_ treatment. The treated sample exhibits a more irregular tube surface in the available SEM images, while the overall conductive framework remains continuous. SEM cannot identify individual chemical groups or resolve atomic-scale surface modification; consequently, no chemical assignment is based on morphology alone. The treatment effect is evaluated primarily from comparative XPS, BET, and electrochemical data. No quantitative electrochemical contribution is inferred from the SEM contrast or apparent surface irregularity.

**Figure 9:**
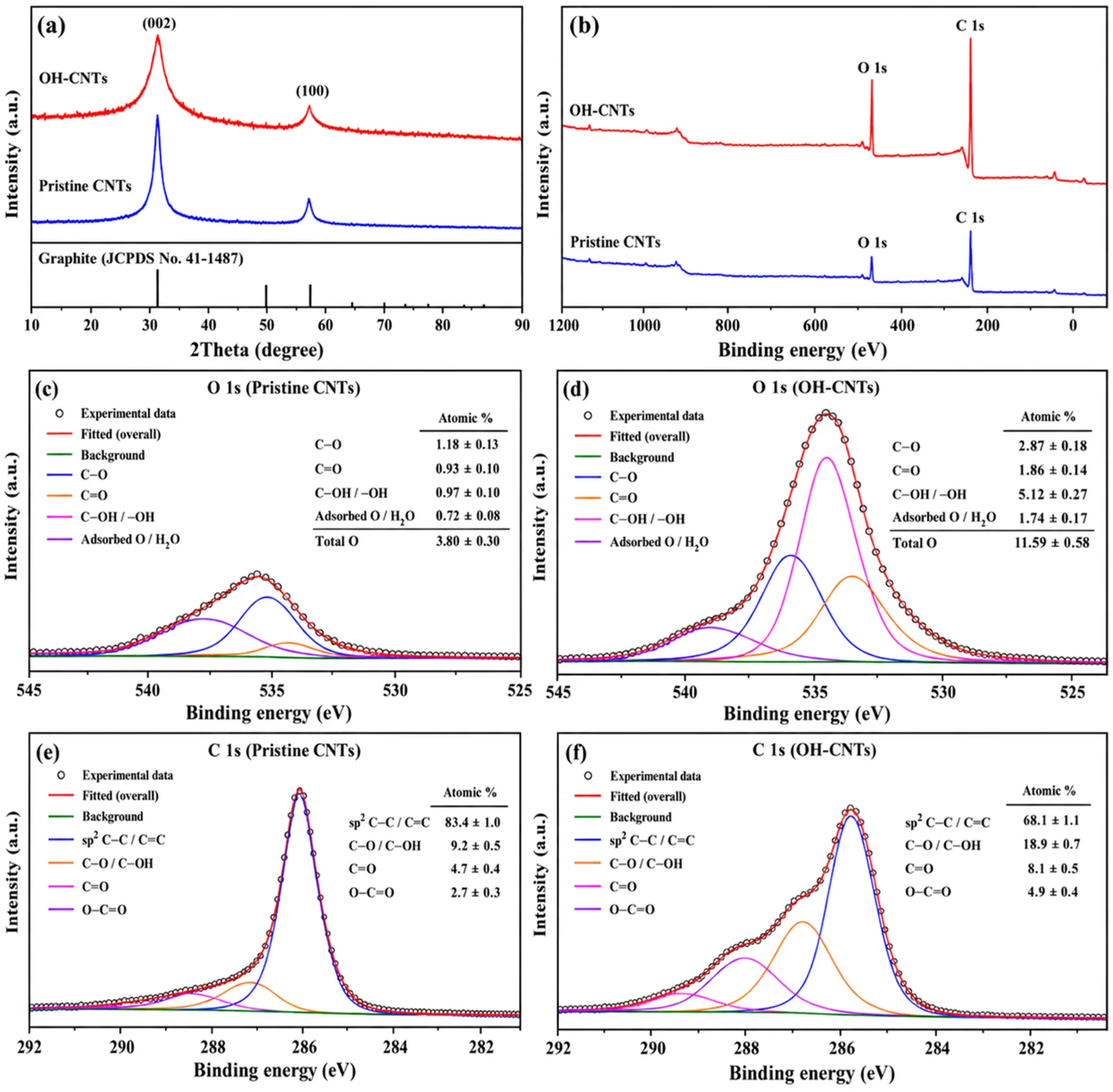
Structural and surface-chemical characterization of pristine CNTs and OH-CNTs. (**a**) XRD patterns. (**b**) XPS survey spectra. (**c**,**d**) O 1s spectra. (**e**,**f**) C 1s spectra. The measured oxygen contents are 3.8 ± 0.3 and 11.6 ± 0.6 at.%, and the BET surface areas are 112 and 138 m^2^ g^−1^ for pristine CNTs and OH-CNTs, respectively.

The XRD patterns in Figure 9a retain the graphitic (002) and (100) reflections after H_2_O_2_ treatment, indicating preservation of the conductive carbon framework. The XPS survey and high-resolution spectra are shown in Figure 9b–f. XPS survey analysis gives oxygen contents of 3.8 ± 0.3 and 11.6 ± 0.6 at.% for pristine CNTs and OH-CNTs, respectively. C 1s fitting gives C–C/C=C, C–O/C–OH, C=O, and O–C=O fractions of 83.4/9.2/4.7/2.7% for pristine CNTs and 68.1/18.9/8.1/4.9% for OH-CNTs. The BET surface area increases from 112 to 138 m^2^ g^−1^ after treatment. The increases in measured oxygen content and BET surface area are consistent with improved aqueous-electrolyte wettability and more effective utilization of the accessible carbon surface. In view of the nearly rectangular CV curves and triangular GCD profiles discussed below, no significant pseudocapacitive contribution is inferred from the oxygen-containing groups.

### 3.9. Electrochemical Performance of OH-CNT Negative Electrodes

**Figure 10:**
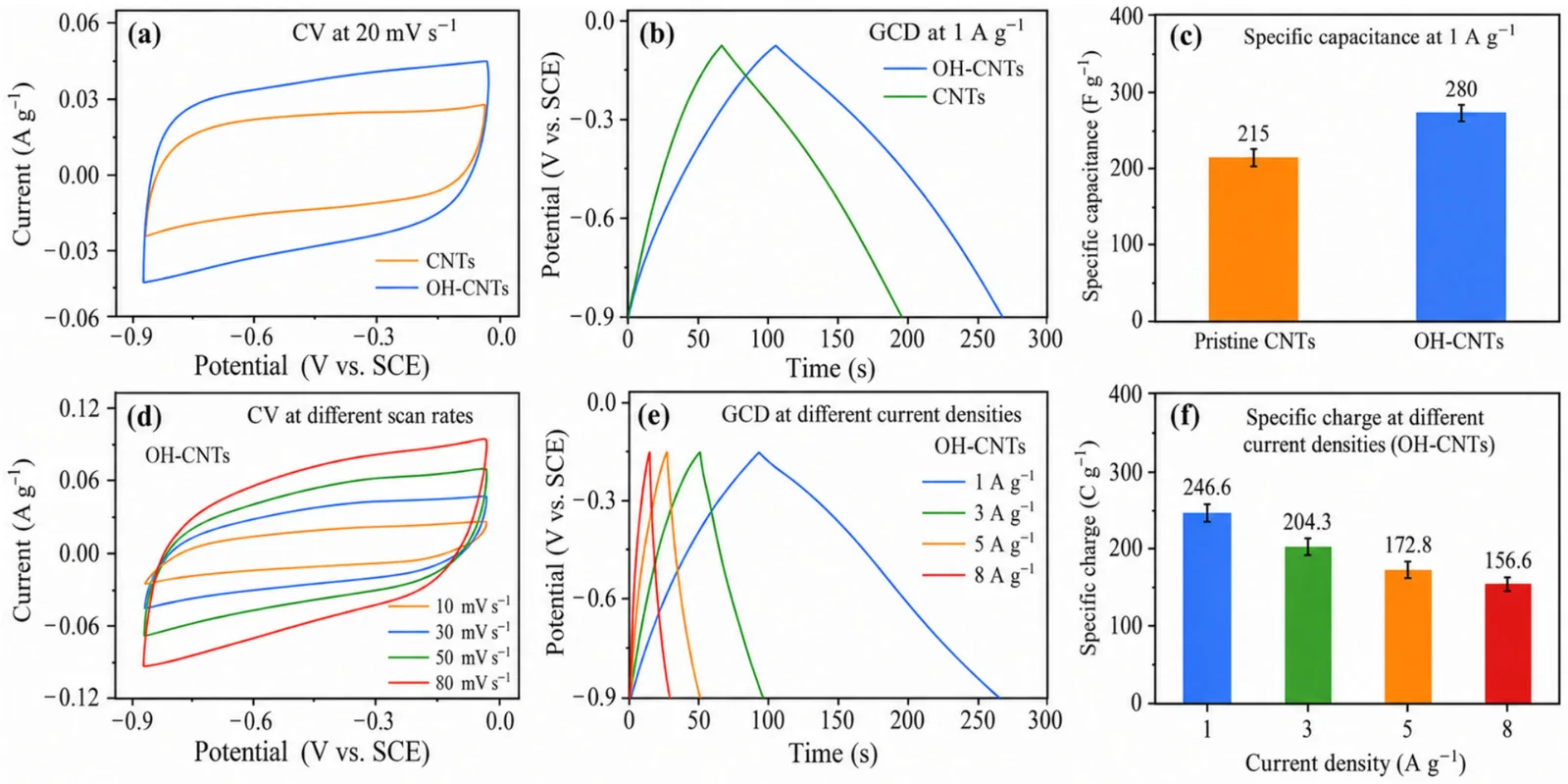
Electrochemical performance of pristine CNT and OH-CNT negative electrodes over −0.9–0 V vs. SCE at active-material loadings of 1.88 ± 0.06 and 1.90 ± 0.05 mg cm^−2^, respectively. (**a**) CV comparison. (**b**) GCD comparison. (**c**) Electrode-level apparent capacitance-equivalent values of 215 ± 8 and 280 ± 9 F g^−1^ at 1 A g^−1^, calculated using the corresponding IR-drop-corrected discharge intervals. (**d**) CV curves of OH-CNTs at different scan rates. (**e**) GCD curves at different current densities. (**f**) Specific-charge rate response of 246.6 ± 8.1, 204.3 ± 7.2, 172.8 ± 6.3, and 156.6 ± 5.4 C g^−1^. Values are mean ± standard deviation (*n* = 3), and the blank-current-collector contribution was subtracted. The capacitance-equivalent values are normalized to CNT active mass; the negative-electrode formulation contained 5 wt% acetylene black and 5 wt% PVDF.

Figure 10a–c presents the electrochemical comparison of pristine CNT and OH-CNT negative electrodes. Both CV and GCD measurements were conducted over the identical negative potential window of −0.9–0 V vs. SCE. At 1 A g^−1^, the current-normalized discharge times were 189.0 ± 7.2 and 246.6 ± 8.1 s for pristine CNTs and OH-CNTs, respectively. Because *Q*_s_ = *I*Δ*t*/*m* and *I*/*m* = 1 A g^−1^, these discharge times numerically reproduce the measured of 189.0 ± 7.2 and 246.6 ± 8.1 C g^−1^. Using the measured IR drops of 22 ± 2 and 18 ± 2 mV gives effective discharge intervals of 0.878 and 0.882 V and corresponding capacitance-equivalent values of 215 ± 8 and 280 ± 9 F g^−1^, respectively. This current–time–mass consistency check, together with three independently prepared electrodes and subtraction of the blank nickel-foam response, supports the internal reproducibility of the reported electrode-level results. The active-material loadings were 1.88 ± 0.06 and 1.90 ± 0.05 mg cm^−2^. No metal compound or conducting polymer was intentionally introduced into either negative electrode; however, each coating contained 5 wt% acetylene black and 5 wt% PVDF, and the primary gravimetric values were normalized to CNT active mass. The values are therefore reported as formulation-dependent, electrode-level apparent capacitances rather than intrinsic capacitances of isolated commercial MWCNT powder. On a total-dry-coating basis, the corresponding values are 193.5 ± 7.2 and 252.0 ± 8.1 F g^−1^ for pristine CNTs and OH-CNTs, respectively.

The BET surface area increased from 112 to 138 m^2^ g^−1^ after H_2_O_2_ treatment, corresponding to a 23.2% increase, whereas the CNT-active-mass-normalized apparent capacitance increased by 30.2%. Dividing the apparent capacitance by the measured BET area gives diagnostic area-normalized responses of 1.92 and 2.03 F m^−2^ (approximately 192 and 203 μF cm^−2^) for pristine CNTs and OH-CNTs, respectively, an increase of only about 5.7%. The close magnitudes of the BET-area increase and the gravimetric-response increase support improved wetting, electrolyte accessibility, and utilization of the carbon surface as the principal explanation, rather than assigning the entire 65 F g^−1^ difference to hydroxyl-group pseudocapacitance. This BET-normalized comparison is a diagnostic cross-check rather than an intrinsic interfacial capacitance, because N_2_-accessible area at 77 K is not identical to electrochemically accessible area in 1 M KOH and the acetylene-black contribution was not separately subtracted. Together with the nearly rectangular CV curves and triangular GCD profiles, the BET-normalized comparison supports the interpretation that charge storage in OH-CNTs is dominated by electric double-layer capacitance. No distinct redox peaks or discharge plateaus are observed; therefore, the present data do not support a significant pseudocapacitive contribution from the oxygen-containing groups.

The rate data in Figure 10d–f provide an additional consistency check: the OH-CNT electrode delivers 246.6 ± 8.1, 204.3 ± 7.2, 172.8 ± 6.3, and 156.6 ± 5.4 C g^−1^ at 1, 3, 5, and 8 A g^−1^, respectively, corresponding to 63.5% specific-charge retention at 8 A g^−1^. Rate-dependent F g^−1^ values are not tabulated because the effective discharge interval changes with current density. Published pure-MWCNT results are not treated as identical-condition validation because CNT source and activation history, residual catalyst content, binder and conductive-additive fraction, loading, current collector, electrolyte, potential window, IR-drop correction, and mass-normalization basis vary substantially among studies. Accordingly, the manuscript does not claim a record or universal intrinsic CNT capacitance; it reports the measured, the explicitly defined apparent capacitance, total-dry-coating and BET-area cross-checks, and replicate uncertainty. The enhanced OH-CNT response is attributed mainly to improved aqueous-electrolyte wettability and more effective utilization of the accessible carbon surface; a significant oxygen-group pseudocapacitive contribution is not invoked. A literature-based comparison with pure and oxygen-modified MWCNT electrodes is provided in Section 3.12; the cited studies are used for context rather than as identical-condition validation.

### 3.10. Charge-Balanced Device Assembly and Electrode Complementarity

**Figure 11:**
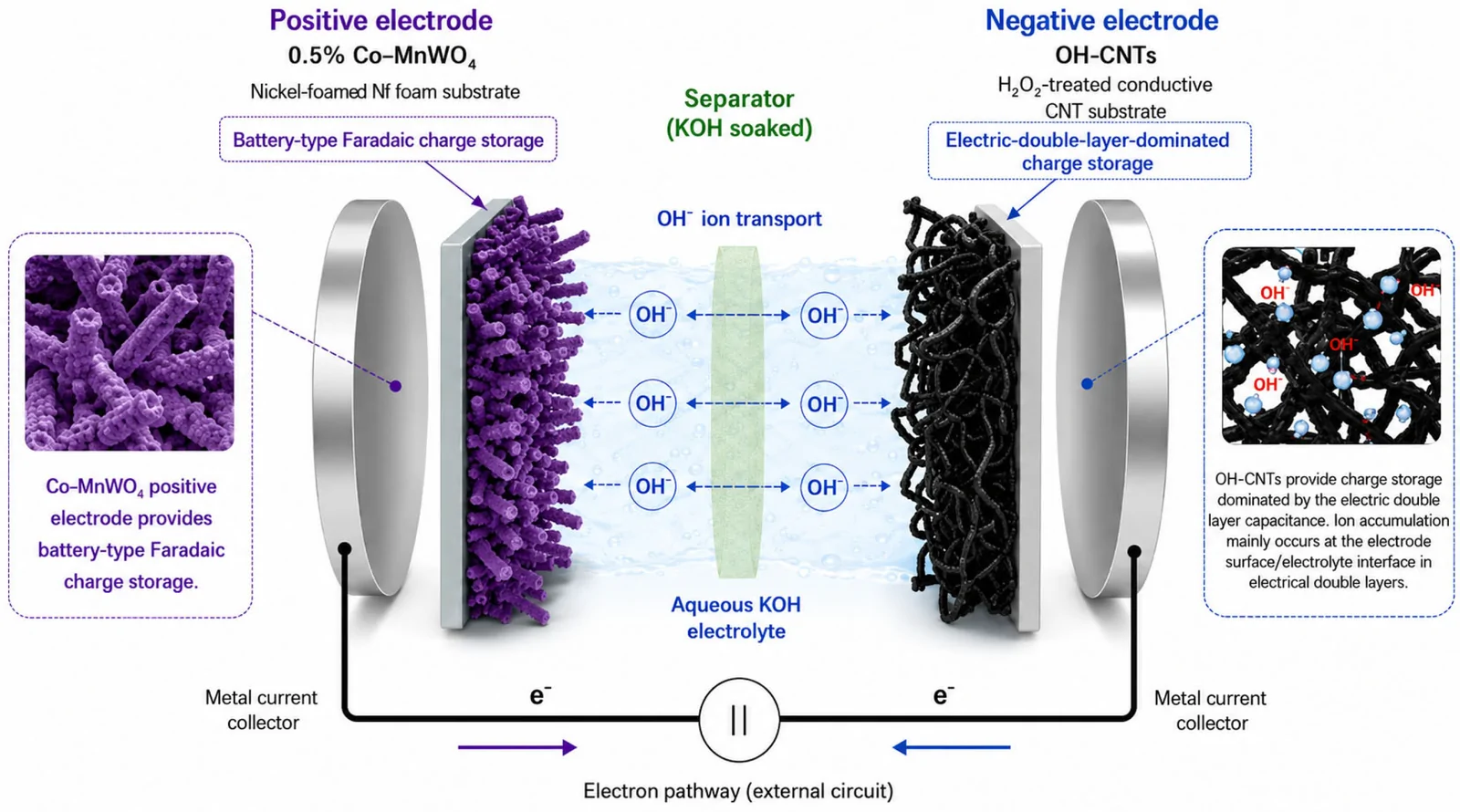
Schematic of the charge-balanced Co-MnWO_4_//OH-CNT aqueous hybrid device. The positive electrode provides battery-type Faradaic charge storage, while OH-CNTs provide charge storage dominated by electric double-layer capacitance. The oxygen-containing groups are shown mainly as improving aqueous-electrolyte wettability and utilization of the accessible carbon surface. The schematic illustrates ionic and electronic pathways and is not presented as direct evidence of a specific interelectrode electronic interaction.

The charge-balanced device architecture and electrode complementarity are summarized in Figure 11. A Co-MnWO_4_//OH-CNT aqueous hybrid device was assembled from electrodes evaluated in their respective positive and negative potential ranges. The nominal 0.5 mol% Co-MnWO_4_ electrode stores charge through reversible battery-type Faradaic reactions over 0–0.5 V vs. SCE, whereas OH-CNTs operate over −0.9–0 V vs. SCE and provide charge storage dominated by electric double-layer capacitance. The oxygen functionalities are considered to improve aqueous-electrolyte wettability and utilization of the accessible carbon surface. Using *Q*_s_^+^ = 429.5 C g^−1^ and *Q*_s_^−^ = 246.6 C g^−1^ gives *m*^+^/*m*^−^ = 0.574. For the 1.0 cm^2^ device, the positive and negative active masses were 2.00 and 3.48 mg. At matched absolute current, the measured *q*^+^/*q*^−^ = 1.008 and confirming effective charge matching. The 1.6 V cell window was selected by stepwise full-cell screening rather than by summing the separately tested three-electrode windows; it was the highest tested device window without an abrupt current upturn, an additional irreversible CV feature, or marked GCD distortion. Because the full cell was not equipped with a reference electrode, this value is treated as an empirical two-electrode operating window and is not used to claim that the instantaneous potential of either electrode remained within its isolated three-electrode range.

The device combines a high-charge Faradaic positive electrode with a conductive and electrolyte-accessible carbon negative electrode. This complementary configuration was designed to reduce charge and kinetic mismatch between the electrodes. The discussion is therefore framed in terms of electrode complementarity rather than a direct electronic interaction between physically separated materials. Related layered-hydroxide and supercapattery studies likewise show that complementary electrodes can improve full-cell output while requiring careful distinction among electric-double-layer, pseudocapacitive, and battery-type contributions [36,37]. Device capacitance, specific energy, and power were calculated from full-cell GCD curves using the combined active mass of both electrodes.

### 3.11. Electrochemical Performance of the Co-MnWO_4_//OH-CNT Hybrid Device

**Figure 12:**
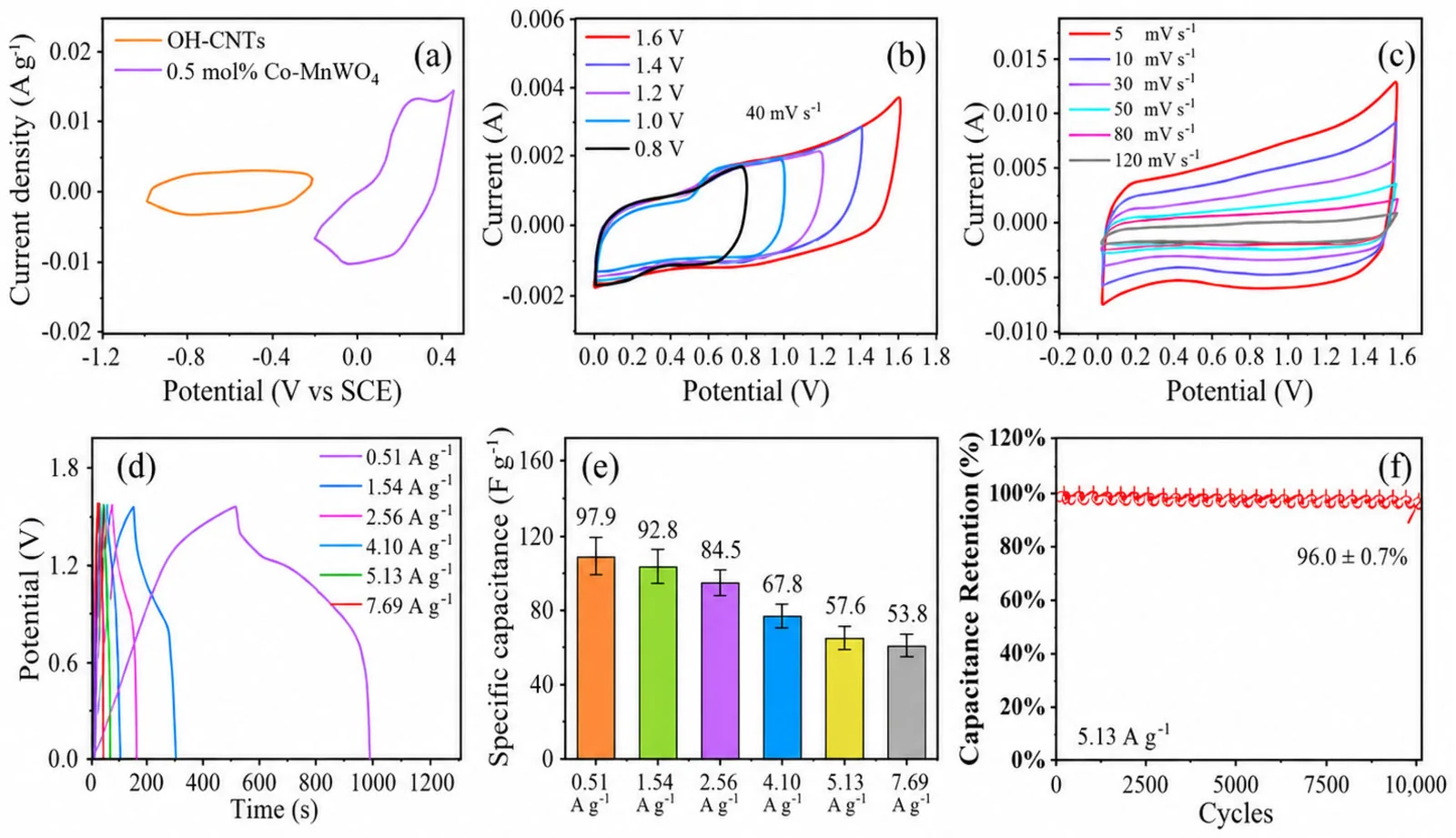
Electrochemical performance of the assembled Co-MnWO_4_//OH-CNT aqueous hybrid device. (**a**) CV curves of the independently evaluated positive and negative electrodes in their respective potential ranges. (**b**) Device CV curves under different voltage windows. (**c**) CV curves at different scan rates over 0–1.6 V. (**d**) GCD curves at combined-active-mass current densities of 0.51–7.69 A g^−1^. (**e**) Device capacitances of 97.9 ± 3.1, 92.8 ± 3.0, 84.5 ± 2.8, 67.8 ± 2.4, 57.6 ± 2.1, and 53.8 ± 2.0 F g^−1^. (**f**) Mean capacitance retention of 96.0 ± 0.7% and coulombic efficiency of 98.8 ± 0.1% at the 10,000th cycle at 5.13 A g^−1^ and 25 ± 2 °C (*n* = 3); coulombic efficiency was calculated as *Q*_discharge_/*Q*_charge_ × 100%. All device metrics are normalized to *m*^+^ + *m*^−^.

Figure 12 summarizes the device response. The individual-electrode CV curves in Figure 12a show complementary operating regions, and the stepwise voltage-window screening in Figure 12b identifies 1.6 V as the highest empirically screened full-cell window under the reported conditions; individual electrode potentials were not resolved in the assembled two-electrode device. The selected window showed no abrupt current upturn or additional irreversible feature in CV and no pronounced gas-evolution-related distortion in the GCD profile. The scan-rate-dependent CV curves in Figure 12c retain similar overall profiles, while increased peak separation and profile distortion at high scan rates indicate growing polarization. This stability assessment applies specifically to the present electrode pair, loading, and 1 M KOH electrolyte and does not establish a universal 1.6 V aqueous stability limit.

The GCD curves in Figure 12d give combined-active-mass device capacitances of 97.9 ± 3.1, 92.8 ± 3.0, 84.5 ± 2.8, 67.8 ± 2.4, 57.6 ± 2.1, and 53.8 ± 2.0 F g^−1^ at 0.51, 1.54, 2.56, 4.10, 5.13, and 7.69 A g^−1^, respectively, as summarized in Figure 12e. The device retains 55.0% of its low-current capacitance at the highest current density. For the reported cycling metric in Figure 12f, coulombic efficiency was calculated as η = *Q*_discharge_/*Q*_charge_ × 100%. Across three independently assembled devices, the values at the 10,000th cycle were 96.0 ± 0.7% capacitance retention and 98.8 ± 0.1% coulombic efficiency after cycling at 5.13 A g^−1^ over 0–1.6 V and 25 ± 2 °C.

**Figure 13:**
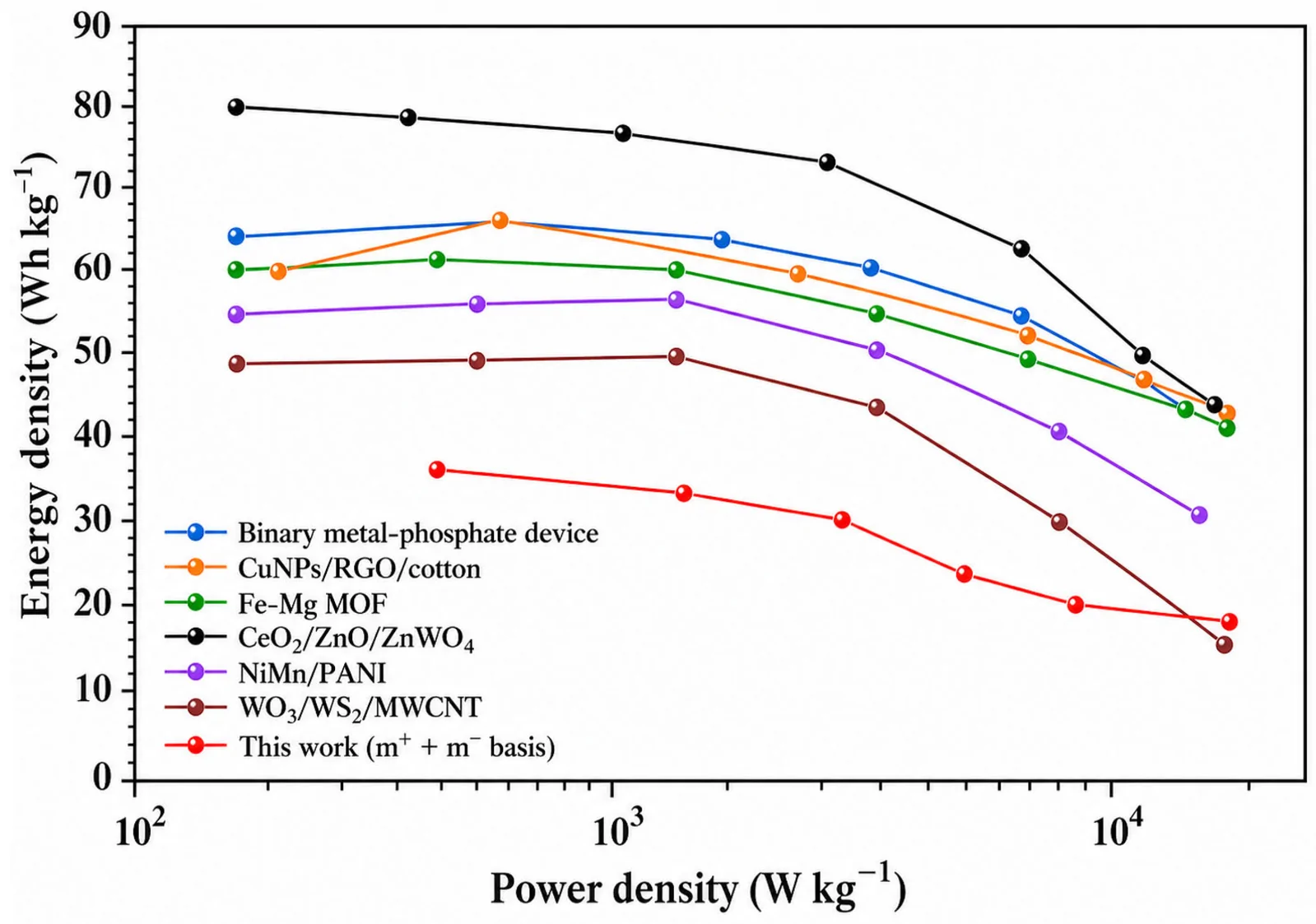
Contextual Ragone plot comparing the Co-MnWO_4_//OH-CNT device with selected aqueous asymmetric or hybrid systems. This work delivers an active-material-level specific energy of 34.8 Wh kg^−1^ at 408 W kg^−1^, normalized to the combined active mass of both electrodes. Literature values are reproduced as reported and are not used for a strict performance ranking because normalization conventions and test configurations differ.

Numerical integration of the full-cell discharge curve gives an active-material-level specific energy of 34.8 Wh kg^−1^ at an average power of 408 W kg^−1^, using the combined active mass of both electrodes. The electrode loadings, test conditions, normalization bases, and principal electrochemical performance metrics are summarized in Table 6. At higher current densities, the corresponding specific energies are 33.0, 30.0, 24.1, 20.5, and 19.1 Wh kg^−1^ at 1232, 2048, 3280, 4104, and 6152 W kg^−1^, respectively. These values exclude current collectors, separator, electrolyte, and packaging and therefore must not be interpreted as packaged-cell metrics. Figure 13 places the result beside selected aqueous asymmetric or hybrid systems [38,39,40,41,42,43] only as a contextual visualization of values reported in the cited articles; differences in electrode loading, voltage range, calculation method, and mass basis preclude strict ranking.

Although the present curve lies below several selected literature series in gravimetric energy density, the principal strengths of this work are different: the positive and negative electrodes were independently quantified and experimentally charge-matched to within 0.8% (*q*^+^/*q*^−^ = 1.008), all principal electrochemical values were reproduced with three independently prepared electrodes or devices, and the assembled aqueous device retained 96.0 ± 0.7% capacitance with 98.8 ± 0.1% coulombic efficiency at the 10,000th cycle. The optimized positive electrode used only 0.47 ± 0.03 mol% measured bulk Co while preserving the MnWO_4_ host phase, and the negative electrode was obtained by a simple H_2_O_2_ treatment of commercial CNTs without an intentionally added metal compound or conducting polymer. The study therefore emphasizes quantitative charge balance, low-level composition control, aqueous safety, cycling reproducibility, and transparent combined-active-mass accounting rather than a record energy-density claim.

### 3.12. Practical Scope and Limitations

The measured ICP-OES compositions, Rietveld refinements, comparative XPS data, replicate EIS fits, and kinetic analysis strengthen the composition–structure–performance relationship. Nevertheless, ICP-OES and powder refinement do not identify a unique Co crystallographic site, and the observed electrochemical improvement should not be attributed to a single electronic mechanism. The low-intensity Co 2p deconvolution is semiquantitative. The specific role of EDTA was not isolated with an EDTA-free control, and atomic-scale surface modification of OH-CNTs was not independently resolved by high-resolution microscopy; the CNT interpretation therefore rests primarily on XPS, BET, and electrochemical comparisons. The nearly rectangular CNT CV curves and triangular GCD profiles indicate that the negative-electrode charge storage is dominated by electric double-layer capacitance; accordingly, the 65 F g^−1^ capacitance-equivalent difference is not assigned to oxygen-group pseudocapacitance. The current–time–mass, total-dry-coating, and BET-area normalizations improve transparency but do not convert the electrode-level apparent capacitance into an intrinsic powder property or replace an identical-condition literature benchmark. The device loadings (2.00 and 3.48 mg cm^−2^) are suitable for laboratory comparison but do not establish performance at commercial areal loading. The 1.6 V operating window applies only to the present electrode pair and electrolyte under the reported conditions; individual electrode potentials were not monitored in the assembled two-electrode device. Acetylene-black contributions were not separately subtracted, and the conservative total-dry-solid normalizations are therefore provided in Section 2.3. Finally, the reported primary specific energy and power values are combined-active-mass metrics and will decrease when conductive additive, binder, current collectors, separator, electrolyte, and packaging are included. These limitations define the scope of the claims without altering the reproducibility within the series.

The available peer-reviewed literature also demonstrates why direct numerical validation of a commercial MWCNT electrode requires caution. Tao et al. reported approximately 33 F g^−1^ for a pure MWCNT electrode tested at 0.5 A g^−1^ in 6 M KOH over −1–0 V, a closely related alkaline potential window but a different electrode formulation, loading, current density, and normalization basis [44]. Mombeshora et al. compared several oxygen treatments, including H_2_O_2_, and showed that the capacitance enhancement of oxygen-modified MWCNTs depended strongly on the oxidant and electrolyte; the reported absolute values in group-one sulfate electrolytes remained substantially dependent on the measurement system [45]. Costa et al. likewise found that improved performance after MWCNT oxidation reflected a balance among accessible surface area, oxygen loading, and electrical conductivity, rather than a universal contribution from one functional group [46]. These reports do not establish an identical-condition benchmark for the present 90:5:5 CNT/acetylene-black/PVDF electrode and therefore are not used to prove the absolute values of 215 or 280 F g^−1^. Instead, they support the restricted interpretation adopted here: CNT source, electrode architecture, electrolyte accessibility, surface chemistry, conductive-additive contribution, and normalization convention jointly determine the measured electrode-level response. A contextual comparison of selected MnWO_4_-based reports and the present work is provided in Table 7.

## 4. Conclusions

Co-modified MnWO_4_ nanorods and H_2_O_2_-treated CNTs were integrated as complementary electrodes in a quantitatively charge-balanced aqueous hybrid supercapacitor. ICP-OES measured 0.47 ± 0.03 mol% Co in the optimized nominal 0.5 mol% sample, while Rietveld refinement confirmed retention of the monoclinic P2/c phase with only small lattice-parameter changes. Within the investigated series, this sample exhibited the largest BET surface area and mesopore volume, the lowest charge-transfer resistance, and the largest surface-controlled kinetic fraction. The battery-type positive electrode delivered 429.5 ± 11.0 C g^−1^ (119.3 ± 3.1 mAh g^−1^) at 1 A g^−1^ and retained 280.5 ± 8.5 C g^−1^ (77.9 ± 2.4 mAh g^−1^) at 15 A g^−1^. H_2_O_2_ treatment increased the measured CNT oxygen content and BET surface area. Under the stated 90:5:5 negative-electrode formulation and CNT-active-mass normalization, the electrode-level apparent capacitance increased from 215 ± 8 to 280 ± 9 F g^−1^. The nearly rectangular CV curves and triangular GCD profiles identify the negative-electrode response as predominantly electric-double-layer capacitance. Consistent with the close correspondence between the relative BET-area and gravimetric-response increases, the oxygen functionalities are considered to improve electrolyte wettability and utilization of the accessible carbon surface rather than to provide a significant pseudocapacitive contribution. A mass ratio of *m*^+^/*m*^−^ = 0.574 produced an experimentally measured charge ratio *q*^+^/*q*^−^ = 1.008. The device delivered 97.9 ± 3.1 F g^−1^ at 0.51 A g^−1^ and a specific energy of 34.8 Wh kg^−1^ at 408 W kg^−1^ when normalized to the combined active mass of both electrodes, with 96.0 ± 0.7% capacitance retention and 98.8 ± 0.1% coulombic efficiency at the 10,000th cycle. Rather than establishing a record Ragone performance, the results demonstrate low-level composition control, explicit electrode charge matching, reproducibility across independent devices, long-term aqueous cycling stability, and transparent mass accounting as complementary laboratory-scale design strategies. Packaged-cell performance, commercial areal loading, a unique Co site, the isolated role of EDTA, and atomic-scale CNT surface morphology remain outside the present scope.

## Figures and Tables

**Table 1:** Calculated peak positions and hkl assignments for monoclinic MnWO_4_ based on the Rietveld-refined P2/c structure.

hkl	Calculated 2θ (°)	Calculated d (Å)
(010)	15.376	5.758
(100)	18.361	4.828
(011)	23.560	3.773
(110)	24.035	3.700
(111)	30.259	2.951
(020)	31.038	2.879
(021)	35.976	2.494
(200)	37.216	2.414
(121)	40.854	2.207
(112)	44.021	2.055
(211)	44.842	2.020
(220)	49.218	1.850
(130)	51.175	1.784
(221)	53.011	1.726
(202)	53.259	1.719
(113)	61.361	1.510
(132)	64.359	1.446
(041)	67.683	1.383

**Table 2:** Quantitative XPS fitting parameters for pristine MnWO_4_ and 0.5 mol% Co-MnWO_4_.

Sample	Component	Binding Energy/Survey at.%
MnWO_4_	Survey (C excluded)	Mn 16.1; W 16.4; O 67.5; Co 0
MnWO_4_	Mn^2+^/Mn^3+^ 2p_3_/_2_	640.30/641.65 eV
MnWO_4_	W^6+^/W^5+^ 4f_7_/_2_	34.90/33.78 eV
MnWO_4_	O 1s: lattice/OH-defect/adsorbed	529.45/531.05/532.55 eV
0.5 mol% Co-MnWO_4_	Survey (C excluded)	Mn 15.9; W 16.2; O 67.8; Co 0.10
0.5 mol% Co-MnWO_4_	Mn^2+^/Mn^3+^ 2p_3_/_2_	640.48/641.82 eV
0.5 mol% Co-MnWO_4_	W^6+^/W^5+^ 4f_7_/_2_	35.02/33.90 eV
0.5 mol% Co-MnWO_4_	O 1s: lattice/OH-defect/adsorbed	529.53/531.16/532.62 eV
0.5 mol% Co-MnWO_4_	Co^3+^/Co^2+^ 2p_3_/_2_	779.65/781.05 eV

**Table 3:** Pore-structure parameters and selected electrochemical descriptors of MnWO_4_ and Co-modified samples.

Sample	BET Surface Area (m^2^ g^−1^)	Total Pore Volume (cm^3^ g^−1^)	Mesopore Volume (cm^3^ g^−1^)	Average BJH Pore Diameter (nm)	Specific Charge at 1 A g^−1^ (C g^−1^)	R_ct_ (Ω)
MnWO_4_	42.6	0.24	0.21	22.5	354.0 ± 9.0	5.9 ± 0.3
0.1 mol% Co-MnWO_4_	55.8	0.28	0.25	20.1	383.0 ± 10.0	4.7 ± 0.2
0.5 mol% Co-MnWO_4_	72.4	0.33	0.30	18.2	429.5 ± 11.0	3.2 ± 0.2
0.8 mol% Co-MnWO_4_	61.7	0.30	0.27	19.4	406.0 ± 10.5	4.0 ± 0.2

**Table 4:** Equivalent-circuit fitting parameters for MnWO_4_ and Co-modified electrodes (mean ± standard deviation, *n* = 3).

Sample	R_s_ (Ω)	R_ct_ (Ω)	CPE-T (Ω^−1^ s^n^)	CPE-P (n)	Warburg Coefficient (Ω s^−1/2^)	Fitting χ^2^
MnWO_4_	2.8 ± 0.1	5.9 ± 0.3	0.021 ± 0.002	0.82 ± 0.02	9.8 ± 0.6	2.1 × 10^−3^
0.1 mol% Co-MnWO_4_	2.6 ± 0.1	4.7 ± 0.2	0.026 ± 0.002	0.84 ± 0.02	8.1 ± 0.5	1.8 × 10^−3^
0.5 mol% Co-MnWO_4_	2.2 ± 0.1	3.2 ± 0.2	0.034 ± 0.003	0.88 ± 0.02	5.6 ± 0.4	1.3 × 10^−3^
0.8 mol% Co-MnWO_4_	2.4 ± 0.1	4.0 ± 0.2	0.029 ± 0.002	0.86 ± 0.02	6.9 ± 0.5	1.6 × 10^−3^

**Table 5:** Charge-storage kinetic parameters and fitting quality of pristine and Co-modified MnWO_4_ electrodes (mean ± standard deviation, *n* = 3). The R^2^ range covers the anodic and cathodic b-value fits, and the corresponding anodic and cathodic peak-current–v^1/2^ fits.

Sample	b (Anodic)	b (Cathodic)	Surface-Controlled at 35 mV s^−1^ (%)	Diffusion-Associated at 35 mV s^−1^ (%)	R^2^ Range (minimum)
MnWO_4_	0.68 ± 0.02	0.62 ± 0.02	54 ± 2	46 ± 2	0.992–0.997 (0.992)
0.1 mol% Co-MnWO_4_	0.74 ± 0.02	0.69 ± 0.02	63 ± 2	37 ± 2	0.993–0.998 (0.993)
0.5 mol% Co-MnWO_4_	0.82 ± 0.02	0.76 ± 0.02	72 ± 2	28 ± 2	0.995–0.999 (0.995)
0.8 mol% Co-MnWO_4_	0.77 ± 0.02	0.71 ± 0.02	66 ± 2	34 ± 2	0.993–0.998 (0.993)

**Table 6:** Electrochemical performance, electrode loading, test conditions, and normalization basis of the present work.

Component	Active-Material Loading	Potential/Voltage Window	Normalization Basis	Specific Capacity/Capacitance/Energy Performance	Rate/Cycling Performance
0.5 mol% Co-MnWO_4_ positive electrode	2.00 ± 0.05 mg cm^−2^ on nickel foam	0–0.5 V vs. SCE	Positive-electrode active mass	429.5 ± 11.0 C g^−1^ (119.3 ± 3.1 mAh g^−1^; 893 ± 23 F g^−1^ IR-drop-corrected equivalent) at 1 A g^−1^	280.5 ± 8.5 C g^−1^ (77.9 ± 2.4 mAh g^−1^) at 15 A g^−1^; 65.3% charge retention; 5000 cycles at 10 A g^−1^
OH-CNT negative electrode	1.90 ± 0.05 mg cm^−2^ (single electrode); 3.48 mg cm^−2^ (device) on nickel foam	−0.9–0 V vs. SCE	Negative-electrode active mass	246.6 ± 8.1 C g^−1^; electrode-level apparent capacitance equivalent of 280 ± 9 F g^−1^ after IR-drop correction at 1 A g^−1^ (CNT-active-mass normalization; 90:5:5 formulation)	156.6 ± 5.4 C g^−1^ at 8 A g^−1^; 63.5% charge retention
Co-MnWO_4_//OH-CNT hybrid device	2.00 mg positive + 3.48 mg negative = 5.48 mg total active mass	0–1.6 V	Combined active mass, m^+^ + m^−^	97.9 ± 3.1 F g^−1^; 34.8 Wh kg^−1^ at 408 W kg^−1^; q^+^/q^−^ = 1.008	96.0 ± 0.7% retention and 98.8 ± 0.1% coulombic efficiency after 10,000 cycles at 5.13 A g^−1^ and 25 ± 2 °C (*n* = 3)

Note: All electrochemical tests were conducted in 1 M KOH. Positive-electrode values are reported primarily as specific charge/capacity because of battery-type behavior; capacitance-equivalent values are secondary descriptors. Negative-electrode values are normalized to the active mass of the corresponding electrode. The reported CNT capacitance-equivalent values are formulation-dependent electrode values and should not be interpreted as intrinsic capacitances of isolated CNT powder. Device capacitance, specific energy, and power are normalized to the combined active mass of both electrodes. Values are mean ± standard deviation from three independently prepared electrodes or devices unless otherwise stated.

**Table 7:** Contextual comparison of selected MnWO_4_-based reports and the present work. The entries are not ranked because the test and normalization bases differ.

System	Test Configuration	Loading/Mass Basis	Reported Metric	Comparability Note
MnWO_4_–amorphous CNT hybrid [12]	Three-electrode; CV at 2 mV s^−1^	Not stated in the present summary	542.18 F g^−1^	CV-derived value; not directly comparable with GCD-derived battery-type capacity
MnWO_4_–CNT [13]	Three-electrode; CV at 10 mV s^−1^	As reported in the cited article	1849.14 F g^−1^	Different loading, window, current collector, and normalization may apply
0.5 mol% Co-MnWO_4_ positive electrode (this work)	Three-electrode; GCD at 1 A g^−1^; 0–0.5 V vs. SCE	2.00 ± 0.05 mg cm^−2^; active-material mass	429.5 ± 11.0 C g^−1^ (119.3 ± 3.1 mAh g^−1^; 893 ± 23 F g^−1^ IR-drop-corrected equivalent)	Battery-type metric reported primarily as charge/capacity
Co-MnWO_4_//OH-CNT device (this work)	Two-electrode; GCD at 0.51 A g^−1^; 0–1.6 V	m^+^ + m^−^ = 5.48 mg	97.9 ± 3.1 F g^−1^; 34.8 Wh kg^−1^ at 408 W kg^−1^	Combined-active-mass basis; excludes inactive components

Note: Literature values are reproduced as cited in the manuscript. Strict quantitative ranking is avoided because CV- and GCD-derived metrics, single-electrode and full-cell tests, and different mass bases are not equivalent.

## Data Availability

The data supporting the findings of this study, including the raw CV, GCD, EIS, XRD, XPS, EDS, and N_2_ adsorption–desorption data, are available from the corresponding author upon reasonable request.

## Abbreviations

The following abbreviations are used in this manuscript:

Abbreviation	Full name	Abbreviation	Full name
BET	Brunauer–Emmett–Teller	BJH	Barrett–Joyner–Halenda
CNT	Carbon nanotube	CPE	Constant-phase element
CV	Cyclic voltammetry	EDS	Energy-dispersive X-ray spectroscopy
EDTA	Ethylenediaminetetraacetic acid	EIS	Electrochemical impedance spectroscopy
GCD	Galvanostatic charge–discharge	HRTEM	High-resolution transmission electron microscopy
ICP-OES	Inductively coupled plasma optical emission spectroscopy	NMP	N-methyl-2-pyrrolidone
OH-CNT	H_2_O_2_-treated oxygen-functionalized carbon nanotube (operational label)	PVDF	Poly(vinylidene fluoride)
SAED	Selected-area electron diffraction	SCE	Saturated calomel electrode
SEM/TEM	Scanning/transmission electron microscopy	XPS/XRD	X-ray photoelectron spectroscopy/X-ray diffraction
